# A least microenvironmental uncertainty principle (LEUP) as a generative model of collective cell migration mechanisms

**DOI:** 10.1038/s41598-020-79119-y

**Published:** 2020-12-22

**Authors:** Arnab Barua, Josue M. Nava-Sedeño, Michael Meyer-Hermann, Haralampos Hatzikirou

**Affiliations:** 1 Department of Systems Immunology and Braunschweig Integrated Centre of Systems Biology, Helmholtz Centre for Infection Research, Rebenring 56, 38106 Braunschweig, Germany; 2 Center for Information Services and High Performance Computing, Technische Univesität Dresden, Nöthnitzer Straße 46, 01062 Dresden, Germany; 3 Institute for Biochemistry, Biotechnology and Bioinformatics, Technische Universität Braunschweig, Braunschweig, Germany; 4 Universidad Nacional Autónoma de México, Faculty of Sciences, Department of Mathematics, Circuito Exterior, Ciudad Universitaria, 04510 Mexico City, Mexico; 5 Mathematics Department, Khalifa University, P.O. Box 127788, Abu Dhabi, UAE

**Keywords:** Biological physics, Statistical physics, thermodynamics and nonlinear dynamics, Computational biophysics

## Abstract

Collective migration is commonly observed in groups of migrating cells, in the form of swarms or aggregates. Mechanistic models have proven very useful in understanding collective cell migration. Such models, either explicitly consider the forces involved in the interaction and movement of individuals or phenomenologically define rules which mimic the observed behavior of cells. However, mechanisms leading to collective migration are varied and specific to the type of cells involved. Additionally, the precise and complete dynamics of many important chemomechanical factors influencing cell movement, from signalling pathways to substrate sensing, are typically either too complex or largely unknown. The question is how to make quantitative/qualitative predictions of collective behavior without exact mechanistic knowledge. Here we propose the least microenvironmental uncertainty principle (LEUP) that may serve as a generative model of collective migration without precise incorporation of full mechanistic details. Using statistical physics tools, we show that the famous Vicsek model is a special case of LEUP. Finally, to test the biological applicability of our theory, we apply LEUP to construct a model of the collective behavior of spherical *Serratia marcescens* bacteria, where the underlying migration mechanisms remain elusive.

## Introduction

Collective movement of dense populations is observed in several biological systems at different scales, from massive migration of mammals^1^ to cells during embryogenesis^2^. In these systems, individuals which are able to propel themselves independently and interact with other nearby, start moving in a coordinated fashion once enough similar individuals are brought together. Due to the relevance of many of these processes to human activity, as well as their pervasiveness, there is a need for quantitative understanding of collective migration. *Mechanistic* models, in particular, incorporate the driving interactions between individuals in the specific system modeled. It is clear that different types of individuals, especially across different spatial scales, synchronize their movements through different mechanisms. This results in a variety of models specific to certain individual species^1,3,4^. In the specific case of biological cells, cellular migration involves numerous biophysical processes such as actin polymerization, receptor recruitment, or in bacteria flagellar motor reversal mechanisms to name a few^5^. However, in many cases the exact knowledge of all participating biophysical/chemical mechanisms related to a particular collective migration pattern is not trivial. Therefore, there is a need to construct models of collective migration which do not require knowledge of the exact interactions between individuals.

To address this situation, several mathematical models introduce a *phenomenological* short-range bias that every individual feels. In one of the most influential collective migration models^6^ the so-called Viscek model, the direction of movement of particles changes towards the mean velocity of individuals in a local neighborhood, inducing long-range swarming at the population level. Such models can be further refined into mechanistic models, where individual particle dynamics are dictated by a system of Langevin equations. In Langevin equation models, the reorientation of individual particle velocities is brought about by the existence of a local interaction potential, which is determined by neighboring particle properties. Collective migration has been achieved, for example, through the introduction of a ferromagnetic-like interaction potential, which locally aligns particle velocities polarly, or a liquid-crystal-like interaction potential, which aligns particle velocities nematically^7^.

**Figure 1:**
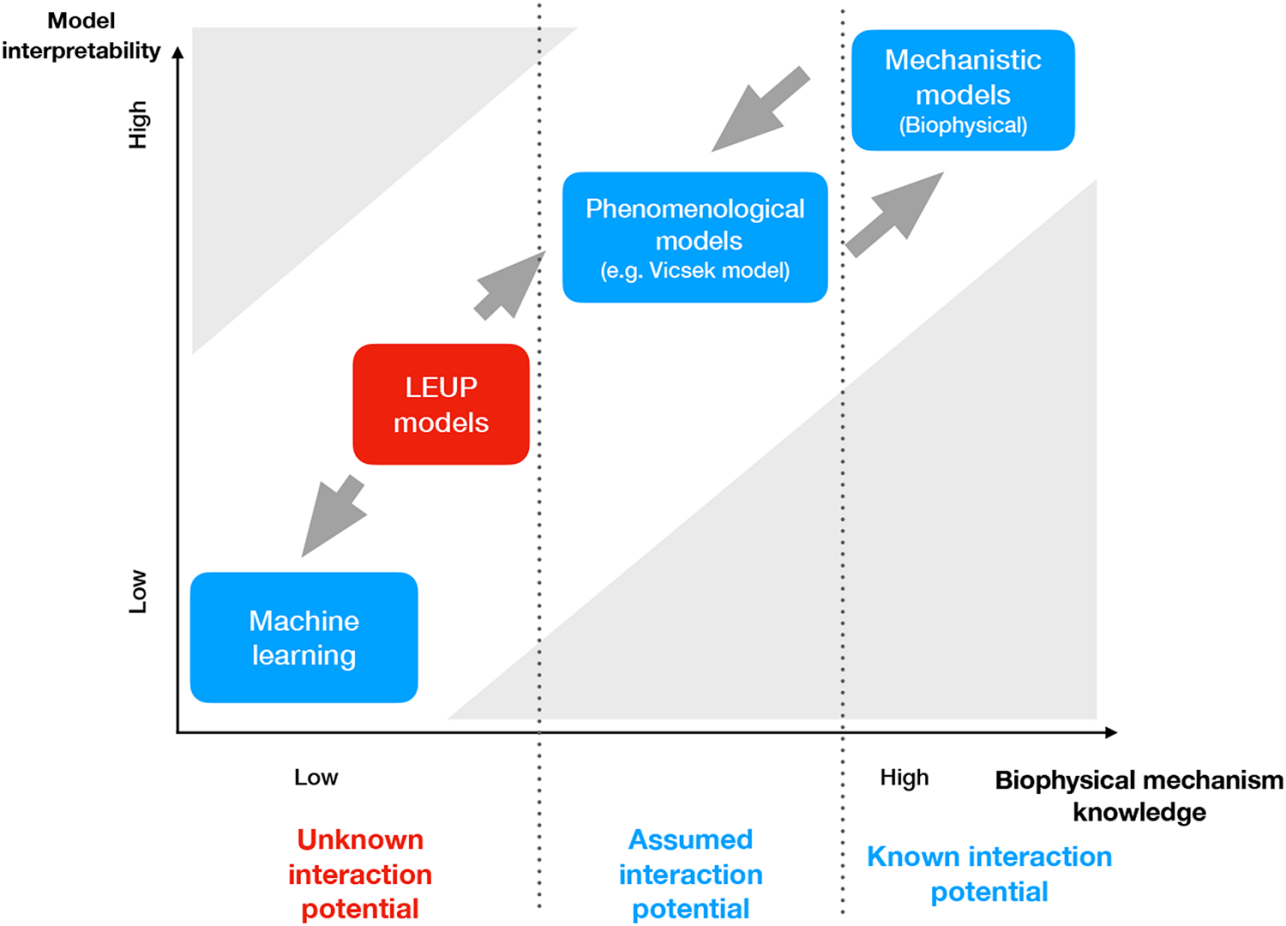
Applicability of different modelling/computational approaches according to their biological interpretability and the corresponding knowledge of biophysical migration mechanisms. The latter is typically encoded as the degree of interaction potential knowledge in Langevin equations (see text). In the case of extended biophysical mechanism knowledge, mechanistic models are the natural choice. When the effects of cell–cell interaction on cell migration are only partially understood, phenomenological models can be typically used. Finally, when data do not suffice to formulate an interaction potential, machine learning allows for the quantitative reproduction of experimental data. However, this has a toll in the interpretability of the resulting model, since machine learning methods are typically “black boxes”. LEUP models offer a compromise that allows for quantitative predictions under lack of mechanism knowledge and satisfactory biological interpretability.

Often neither biophysical nor phenomenological models are able to provide a plausible explanation or quantitative reproduction of collective migration patterns, due to the lack of complete mechanistic knowledge. Such an example is the spatiotemporal dynamics of spherical *S. marcescens* bacteria. Interestingly, prior modeling works^8^ were able to partially reproduce the experimental results, since the underlying biophysical mechanisms are still unclear. In such cases, one could rely of machine/statistical learning methods that circumvent the biophysical details^9–11^. However, such methods are typically of high accuracy but low interpretability, i.e. they are “black boxes” that do not offer mechanistic insights, and prone to overfitting.

Here, we view migration as an active decision-making process. Cell decision-making is the process of cells changing their phenotype according to their intrinsic programming and in response to the microenvironmental cues^12–15^. Cell decisions involve complex biochemical regulation in the genetic, epigenetic, translational or transcriptional level. The fundamental challenges are the (i) uncertainty of high-dimensional subcellular regulatory cell decision-making mechanisms, and the (ii) the lack of knowledge in the relative contribution of intrinsic and extrinsic cell decision-making factors to multicellular spatiotemporal dynamics. Regarding cells as Bayesian decision-makers under energetic constraints, it has been proposed that cell decisions are deictated by a ‘Least microEnvironmental Uncertainty Principle (LEUP)’. This is translated into a free-energy principle, implying a statistical mechanics theory for cell decision-making. Such a statistical mechanics reduction allows for simplifying many parameters into a low-dimensional mathematical description and circumvent the uncertainty about the underlying mechanisms. Moreover, it allows to integrate heterogeneous data types as constraints of LEUP energy optimization. Applying the LEUP to collective cell migration, we aspire (i) to provide a low-dimensional statistical mechanics description, (ii) circumvent the uncertainty about the underlying biophysical mechanisms and (iii) provide a relationship to phenomenological models (e.g. the Vicsek model). Finally in Fig. 1, we illustrate how LEUP is positioned in terms of model interpretability and knowledge of biophysical details in comparison the afore-mentioned modelling/computational approaches. Interestingly, LEUP proposes a balanced solution for problems of low mechanistic knowledge and satisfactory interpretability.

In this work, we present the simplest LEUP-driven Langevin model of swarming where individuals can sense the velocity orientations of other individuals in their surroundings. Individuals act as Bayesian inferrers and change their own orientation to optimize their prior, according to environmental orientation information. Under these assumptions, individuals reorient according to the entropy gradient of the environmental information. A parameter, named the sensitivity, controls the strength and directionality of the reorientation in relation to the local gradient. We find that the system adopts a steady, polar-ordered state for negative values of the sensitivity. Conversely, the system remains out of equilibrium, but partially nematic-ordered when the sensitivity is positive. Furthermore, we find that the qualitative behavior of the model depends on the values of the particle density, noise strength, sensitivity, and size of the interaction neighborhood. Finally, we showcase the LEUP principle by showing that our model replicates the collective behavior of spherical *S. marcescens* bacteria.

## Materials and methods

### The self-propelled particle framework

**Figure 2:**
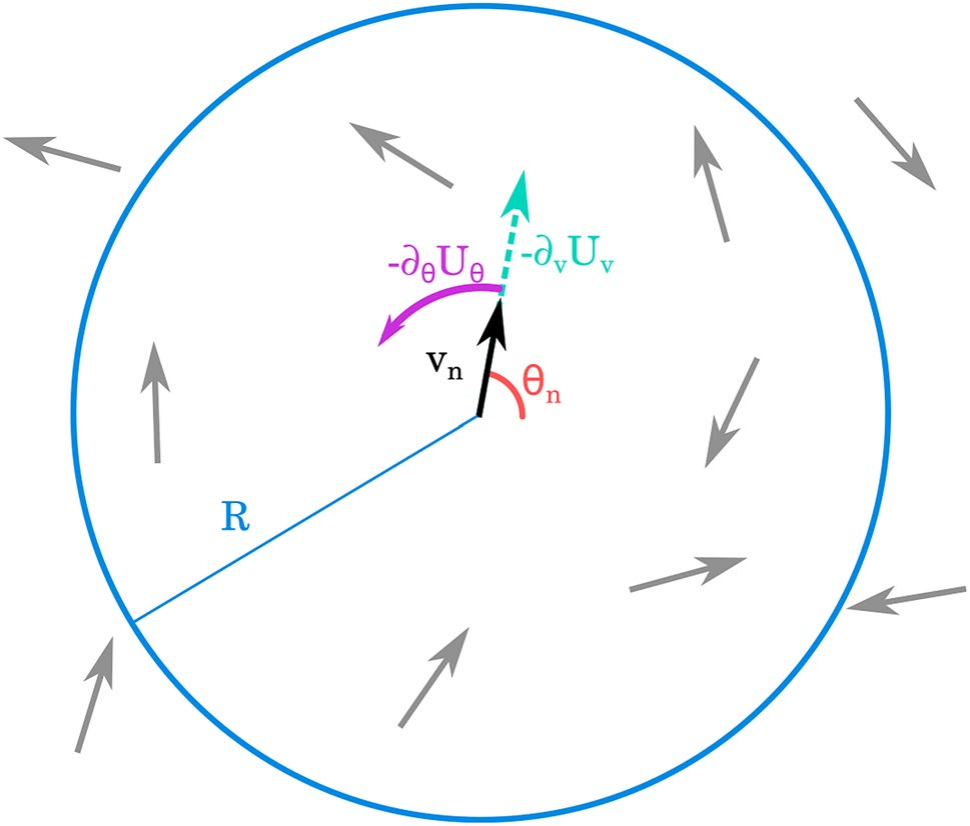
Graphic representation of the dynamics of the SPP model. The *n*-th cell is represented by a point particle with speed $v_n$ and orientation $\theta _n$. Depending on the form of the interaction potential, the cell may feel a reorientation force $-\partial _{\theta }U_{\theta }$ and a radial force $-\partial _vU_v$ due to interaction with other cells inside the interaction neighborhood defined by the radius *R*.

Moving and interacting cells are modeled by a two-dimensional self-propelled particle model (SPP). In this model, $N\in \mathbb {N}$ cells move on a two-dimensional area. The *n*-th cell is characterized by its position, $\vec {r}_n\in \mathbb {R}^2$, speed, $v_n\in [0,\infty )\subset \mathbb {R}$, and an orientation $\theta _n\in [0,2\pi )\subset \mathbb {R}$. Due to the small size of cells, it is assumed that viscous forces dominate. Changes in speed and orientation result from local potentials $U_{\theta }(\vec {r}_m,\theta _m,v_m),U_v(\vec {r}_m,\theta _m,v_m):\mathbb {R}^2\times [0,2\pi )\times [0,\infty )\mapsto \mathbb {R}$ which depend on the positions and polar velocity components of cells within a radius $R\in \mathbb {R}_+$. The bias of the cell to follow the potential gradients are regulated by the parameters $\beta _{\theta },\beta _v\in \mathbb {R}$, called angular and radial sensitivities, respectively. Additionally, velocity fluctuations occur due stochastic noise terms $\xi ^{\alpha }_n(t)\in [0,2\pi )$, $\alpha \in \{\theta ,v\}$ where $t\in \mathbb {R}_+$ denotes time. The noise will be assumed to be a zero-mean, white noise term, which has the statistical properties $\langle \xi ^{\alpha }_n(t)\rangle =0$ and $\langle \xi ^{\alpha }_n(t_1)\xi ^{\alpha }_m(t_2)\rangle =2D_{\alpha }\delta (t_1-t_2)\delta _{nm}$, where $t_1$ and $t_2$ are two time points, $D_{\alpha }\in \mathbb {R}_+$ is either the angular ($\alpha =\theta$) or radial ($\alpha =v$) diffusion coefficient, $\delta (t)$ is the Dirac delta, and $\delta _{nm}$ is the Kronecker delta. Finally, the radial acceleration will be assumed to be damped by a density dependent friction, $\psi (\rho _n)$. In the following, it will be assumed that the density-dependent friction is given by $\psi (\rho _n)=\rho _n-\bar{\rho }$, where $\rho _n$ is the local cell density within the *n*-th cell’s interaction radius, and $\bar{\rho }$ is the global average cell density. Taking everything into account, the stochastic equations of motion of the *n*-th cell read^16^

1a $$\begin{aligned}&\frac{{\mathrm{d}}}{{\mathrm{d}}t}\vec {r}_n=v_n\vec {v}(\theta _n) \end{aligned}$$

1b $$\begin{aligned}&\frac{{\mathrm {d}}}{{\mathrm {d}}t}\theta _n=-\beta _{\theta }\frac{\partial }{\partial \theta _n}U_{\theta }(\vec {r}_m,\theta _m,\vec {v}_{m})+g(\vec {v}_{n})\xi ^{\theta }_n(t) \end{aligned}$$

1c $$\begin{aligned}&\frac{{\mathrm {d}}}{{\mathrm {d}}t}v_n=-\beta _{v}\frac{\partial }{\partial v_n}U_v(\vec {r}_m,\theta _m,\vec {v}_{m})-\varepsilon \psi (\rho _n) v_{n}+\xi ^v_n(t). \end{aligned}$$

where $\vec {v}(\theta _n)$ is the normalized velocity of the cell and $\varepsilon$ is a parameter. A representation of the SPP model is shown in Fig. 2. The function $g(\vec {v}_{n})$ modulates the noise variance and allows us to model certain distributions (such as the Rayleigh distribution in Section 5).

The interaction potentials $U_{\alpha }(\vec {r}_m,\theta _m,\vec {v}_{m})$, which dictate the velocity dynamics of cells, need to be specified. Biophysically, the potentials should encompass steric effects, hydrodynamic interactions, chemotactic effects, and terms arising from internal cellular processes, for example, flagellar motor dynamics, actin polymerization, receptor dynamics, etc. Finding such potentials is a formidable task since not all of the mechanisms and interactions involved are known. To circumvent this problem, a variational principle of cell decision-making related to entropy maximization^17^, known as the least microenvironmental uncertainty principle (LEUP), will be used^18^. In the next section, we will discuss such a case.

### Least microenvironmental uncertainty principle (LEUP)

The main premise of LEUP is that cells equip Bayesian inference to decide their internal states, expressed as a combination of a sensed microenvironmental distribution (empirical/measured likelihood distribution) and a local entropy-dependent prior. From the cellular point of view, constructing an accurate microenvironmental sensing distribution is expensive, since cell sensing is an energetically costly process. Cells sense (collect information) of their surroundings by employing different processes, such as polymerizing pseudopodia, translocating receptor molecules or modifying its cytoskeleton according to mechanical signals^19,20^. However, the cost can be minimized when cells build informative priors about their microenvironment. In the case of moving cells this could be achieved by, for example, promoting cell polarization through the recruitment of actin related proteins^21^. In turn, this allows cells to spare the energy from building extra sensory processes. At same time, cell polarization effectively promotes the co-evolution of the cellular state and its microenvironment, where the latter becomes more predictable. The simultaneous determination of cell state and its microenvironment results in the minimization of the microenvironmental uncertainty towards a target value related to a given tissue or bacteria (e.g. biofilm). This process also is prominent during differentiation, where pluripotent progenitors generically sense, with the goal to find an appropriate differentiation niche, but differentiated cells have very targeted sensors, e.g. precise receptors, that detect a narrow range of tissue-relevant cues. This gives them an advantage since they can invest their energy in optimizing their actual function rather than environmental sensing. The state of the *n*-th cell in this case is defined by its orientation $\theta _n$ and velocity $v_n$. We assume that the orientation and velocity of cells are decoupled, i.e. one can consider orientations and velocities independent from one another. The set of intrinsic angular states of other cells within its radius of interaction is given by $\Theta _n=\{\theta _m:\Vert \vec {r}_n-\vec {r}_m\Vert \le R\}$, while the set of intrinsic velocity states is $V_n=\{v_m:\Vert \vec {r}_n-\vec {r}_m\Vert \le R\}$. The cell reacts to the environmental information, $\Theta _n$ and $V_n$, by changing its own states, $\theta _n$ and $v_n$. The cell then acts as a Bayesian decision-maker, such that

$$\begin{aligned} P (\alpha _n\mid A_n )=\frac{P(A_n\mid \alpha _n)P (\alpha _n )}{P (A_n)}, \end{aligned}$$

$\alpha _n\in \{\theta _n,v_n \}$, $A_n\in \{\Theta _n,V_n \}$ where $P(A_n\mid \alpha _n)$ can be interpreted as the accuracy with which a cell can sense other cells in their surroundings and react accordingly, and $P(\alpha _n)$ is the probability distribution of the cell’s intrinsic states (or prior). However, sensing other cells and evaluating $P(A_n\mid \alpha _n)$ entails an energy cost. It is reasonable to assume that the cell will try to optimize its prior $P(\alpha _n)$ for the sake of energetic frugality.

The prior probabilities $P(\alpha _n)$ should, consequently, fulfill certain the premises of LEUP. First and foremost, they should be normalized, i.e. $\int P(\alpha _n=a){\mathrm {d}}a=1$, integrating over all possible values of internal cellular states. Second, a biological cell is an imperfect sensor. Therefore, the uncertainty in sensing accuracy $S(A_n\mid \alpha _n)$, should reach a certain level in average, which is species-dependent. This assumption is a cornerstone of the LEUP formalism. An important feature of the LEUP formalism, and of the entropy maximization principle in general, is its applicability when every other mechanistic details of the system are unknown. Entropy is a measure of uncertainty according to information theory, therefore entropy should be maximal, in order to reflect our lack of mechanistic knowledge of the phenomenon, and to avoid introducing any artificial bias in the model arising from the specific choice of $P(\alpha _n)$^22^. Given that the internal entropy is given by $S(\alpha _n)=-\int P(\alpha _n=a)\ln P(\alpha _n=a){\mathrm {d}}a$, entropy maximization subjected to probability normalization and the target mean sensing accuracy translates into the optimization problem

2 $$\begin{aligned} &{\frac{\delta }{\delta P(\alpha _n)}} \left\{ -\int P(\alpha _n=a)\ln P(\alpha _n=a){\mathrm {d}}a-\tilde{\beta }_{\alpha }\left[ \int P(\alpha _n=a)S(A_n\mid \alpha _n=a){\mathrm {d}}a-\bar{S}(A_n\mid \alpha _n)\right] \right. \\&\quad \left. -\lambda \left[ \int P(\alpha _n=a){\mathrm {d}}a-1\right] \right\} =0, \end{aligned}$$

where $\frac{\delta }{\delta P(\alpha _n)}$ is the functional derivative, $\bar{S}(A_n\mid \alpha _n)$ is the target sensing accuracy, and $\lambda$ and $\tilde{\beta }_{\alpha }$ are Lagrange multipliers. Taking into account the relations among entropy and probability, Eq. (2) yields

3 $$\begin{aligned} P ( \alpha _{n} = a ) = \frac{e^{-\tilde{\beta }_{\alpha } S ( A_{n} \mid \alpha _{n} = a )}}{Z}, \end{aligned}$$

where $Z = \int e^{-\tilde{\beta }_{\alpha } S(A_{n} \mid \alpha _{n}=a)} {\mathrm {d}}a$ is a normalization constant and $\tilde{\beta }_{\alpha }$ is the responsiveness of the cell. Using Eq. (3), the internal entropy of the cell, defined as $S (\alpha _{n} )=-\int P( \alpha _{n}=a ) \ln P(\alpha _{n} = a ) {\mathrm {d}}a$, is given by

4 $$\begin{aligned} S (\alpha _{n}) = \tilde{\beta }_{\alpha } \overline{S (A_{n} \mid \alpha _{n})} + \ln Z. \end{aligned}$$

Using the relation between thermodynamic-like potentials, it is evident that the average internal energy is given by

5 $$\begin{aligned} U(A_n,\alpha _n)=\overline{S(A_n \mid \alpha _n)} =\langle S(A_{n}\mid \alpha _{n}\ne \alpha )\rangle \end{aligned}$$

and for a particular realisation of $\alpha _n$ we can write the internal energy as

6 $$\begin{aligned} U_{\alpha }(A_n,\alpha _n)=S(A_n\mid \alpha _n) \end{aligned}$$

Now, Helmholtz-like free energy can be written as

7 $$\begin{aligned} F=-\frac{1}{\tilde{\beta }_{\alpha }}\ln Z. \end{aligned}$$

Finally, we return back to the Bayesian formalism and to the main LEUP premise that cells tend to build informative microenvironmental priors $P(\alpha _n)$. In the steady state, the latter implies that

8 $$\begin{aligned} P(\alpha _n|A_n)\xrightarrow {t\rightarrow \infty }P(\alpha _n), \end{aligned}$$

i.e. the posterior distribution of $P(\alpha _n|A_n)$ becomes independent of measuring/sensing the microenvironmental information $A_n$, since the prior $P(\alpha _n)$ includes all relevant information. Observing the Bayesian probability, this only happens when the measurement/sensing likelihood $P(A_n|\alpha _n)$ approaches the probability distribution of the microenvironment $P(A_n)$, i.e. the cell perfectly senses its microenvironment. Using again the Bayesian probability, we can prove the following information-theoretic equation

9 $$\begin{aligned} I(\alpha _n,A_n)= S(\alpha _n)-S(\alpha _n|A_n)\xrightarrow {t\rightarrow \infty } 0. \end{aligned}$$

Substituting the above in Eq. (4), the mutual information reads

10 $$\begin{aligned} I(\alpha _n,A_n)=\tilde{\beta }_{\alpha } \overline{S(A_n \mid \alpha _n)}+\ln Z -S(\alpha _n|A_n). \end{aligned}$$

Two cases have to be considered, depending on the sign of $\tilde{\beta }_{\alpha }$:

- When $\tilde{\beta }_{\alpha }<0$ the only positive term is $\ln Z$ in Eq. (10). Thus, it is reasonable to expect the mutual information to decrease to a minimum value.
- When $\tilde{\beta }_{\alpha }\ge 0$ one can show that the system, for a large enough sensing radius, goes to an ordered state at the steady state (see “Collective cell migration patterns for different parameter regimes”). This implies that the microenvironmental entropy $S(A_n|\alpha _n)$ tends to zero. In this case, the Eq. (10) becomes
  $$\begin{aligned} I(\alpha _n,A_n)=\ln Z -S(\alpha _n|A_n). \end{aligned}$$
  This implies that the mutual information has the potential to reach zero. Interestingly, if this happens using Eq. (9), we can recover a similar definition to Boltzmann entropy for the internal cell state entropy, i.e.
  $$\begin{aligned} S(\alpha _n)=\ln Z. \end{aligned}$$

### LEUP-based dynamics

The internal energy depends on the internal states of the cell, as well as the internal states of other cells in the surroundings. Such internal states can be a vector of physical quantities (e.g. velocity, acceleration) and/or chemical variables such as intracellular proteins, genes and so on. Here, we focus on the former to define an interaction potential that models the equations of motion.

By doing so, it is evident that the responsiveness of the cells to LEUP can be quantified by the sensitivity $\beta _{\alpha }=-\tilde{\beta }_{\alpha }$. Analogously, we can write the equations of motion of the model similar to Eq. (1c) as 

11a $$\begin{aligned}&\frac{{\mathrm {d}}}{{\mathrm {d}}t}\vec {r}_n=v_n\vec {v}(\theta _n) \end{aligned}$$

11b $$\begin{aligned}&\frac{{\mathrm {d}}}{{\mathrm {d}}t}\theta _n=\beta _{\theta }\frac{\partial }{\partial \theta _n}S(\Theta _n\mid \theta _n)+g(\vec {v}_{n})\xi ^{\theta }_n(t) \end{aligned}$$

11c $$\begin{aligned}&\frac{{\mathrm {d}}}{{\mathrm {d}}t}v_n=\beta _{v}\frac{\partial }{\partial v_n}S(V_n\mid v_n)-\varepsilon \psi (\rho _n) v_{n}+\xi ^v_n(t). \end{aligned}$$

To illustrate entropy calculation, it will be assumed that the orientations of cells within the interaction neighborhood are distributed according to

12 $$\begin{aligned} P(\vartheta \in \Theta _n\mid \theta _n)=\frac{\sinh \gamma }{2\pi [\cosh (\gamma )-\cos (\vartheta -\mu )]}, \end{aligned}$$

where $\mu$ is the mean of the distribution and $\gamma$ is a parameter related to the variance. This is a wrapped Cauchy distribution, periodic over the interval $[0,2\pi ]$. Similarly, cell speeds will be assumed to be distributed half-normally

13 $$\begin{aligned} P(v\in V_n\mid v_n)=\sqrt{\frac{2}{\sigma ^2\pi }}\exp \left( -\frac{v^2}{2\sigma ^2}\right) , \end{aligned}$$

where $\sigma ^2$ is proportional to the variance of the distribution. Accordingly, the angular entropy is

14 $$\begin{aligned} S(\Theta _n\mid \theta _n)=\ln (2\pi )+\ln (1-e^{-2\gamma }), \end{aligned}$$

while the speed entropy is

15 $$\begin{aligned} S(V_n\mid v_n)=\frac{1}{2}\ln \left( \frac{\pi \sigma ^2}{2}\right) +\frac{1}{2}. \end{aligned}$$

The parameter $\sigma$ can be determined from the local speed variance, while the parameter $\gamma$ depends on the local polar order (i.e. the degree of parallel alignment) of cell velocities in the neighborhood. It should be noted that the qualitative behavior of the model is independent of the particular choice of distributions, and the distributions considered here are suggested only for ease of calculation. Before defining $\gamma$, we will first define the observables characterizing the order of the velocity field.

### Collective migration observables

Let us define the normalized complex velocity of the *n*-th cell, $z_n\in \mathbb {C}$ as $z_n=e^{i\theta _n}$, where *i* is the imaginary unit. The *k*-th moment of the velocity over an area *A* is given by $\langle z^k\rangle _A=\frac{1}{N_A}\sum _{m\in A}z_m^k$, where the sum is over all cells in area *A*, and $N_A$ is the total number of cells in *A*. The polar order parameter in the area *A* is given by

16 $$\begin{aligned} S^1_A=| \langle z \rangle _A |, \end{aligned}$$

which is the modulus of the first moment of the complex velocity in *A*, while the nematic order parameter in the area *A* is given by

17 $$\begin{aligned} S^2_A=| \langle z^2 \rangle _A |, \end{aligned}$$

which is the modulus of the second moment of the complex velocity in *A*. The order parameters are bounded, i.e.

18 $$\begin{aligned} 0\le S^1_A,S^2_A\le 1, \end{aligned}$$

due to the complex velocities $z_n$ being normalized. The parameter $\gamma$ for the distribution of orientations in the neighborhood of the *n*-th cell is given by

19 $$\begin{aligned} \gamma =-\ln \left( S^1_{C_{R,n}}\right) , \end{aligned}$$

where the subindices $C_{R,n}$ indicate a circular area of radius *R* centered at $\vec {r}_n$. The latter directly stems from the properties of the wrapped Cauchy distribution.

While global polar and/or nematic order are characteristic of steady flows, rotating flow fields are commonly observed in out-of-equilibrium systems. The vorticity is an observable which is equal to twice the local angular velocity, and is thus a measure of the local strength and direction of rotation of the field. The vorticity $\omega$ is defined as

20 $$\begin{aligned} \omega (\vec {r})=[\nabla \times \vec {v}_{{\mathrm {mean}}}(\vec {r})]\cdot \vec {k}, \end{aligned}$$

where $\vec {v}_{{\mathrm {mean}}}(\vec {r})$ is the mean velocity field at point $\vec {r}$, and $\vec {k}$ is vector normal to the plane where cells move.

### Statistical evaluation of experiments and model predictions

For the statistical evaluation of the results we have used the $\chi ^2$-test. The testing hypothesis is that the experimental data are explained by the model predictions. To test it, we construct

21 $$\begin{aligned} \chi _j^2=\sum _{i=1}^{N}\left( \frac{\hat{O}_i^{(j)}-O_i^{(j)}}{\hat{\sigma }_i^{(j)}}\right) ^2, \end{aligned}$$

where $O_i^{(j)}$ is the experimental values of a certain observable j, being either speed or vorticity, and the $\hat{O}_i^{(j)}$ and $\hat{\sigma }_i^{(j)}$ is the corresponding mean value of the stochastic model predictions, based on an ensemble of 50 simulations for each density point $i=1,\ldots ,N,\,N=11$. The quantity $\frac{\hat{O}_i^{(j)}-O_i^{(j)}}{\hat{\sigma }_i^{(j)}}$ can be viewed as a z-score for each $\hat{O}_i^{(j)}$ and for large enough simulation ensemble should converge to a normal distribution. The total degrees of freedom for both observables is $2N=22$. The we calculate the reduced $\chi ^2-$statistic, or $\chi ^2$ per degree of freedom, which is defined as $\chi ^2_{2N}=(2N)^{-1}\sum _j \chi _j^2=1.97$ being close enough to 1. This suggests that our fitting is satisfactory, since values $\chi ^2_{2N}\gg 1$ indicate a bad fit to the experimental data.

## Results

### From LEUP to phenomenological models of collective migration: the relationship to the Vicsek model

By using the LEUP, we have modeled interaction as a change in velocity dictated by the local entropy gradient. The modulation of $\beta _{\alpha }$ parameters modulates the response of cells to the local entropy gradient and gives rise to relationships with known phenomenological models, such as the Vicsek model. The absolute value $| \beta _{\alpha }|$ is proportional to the likelihood of the cell to change its velocity according to a given entropy gradient. If $\beta _{\alpha }<0$, cells tend to go against the local entropy gradient towards the entropy minimum. In the specific case of $\alpha =\theta$, a negative sensitivity would restrict the distribution of angles to a narrow selection. Conversely, $\beta _{\alpha }>0$ forces cells to follow the entropy gradient towards the entropy maximum, broadening the distribution. From here on, we will assume that the effect of cell interactions will be averaging the radial component, therefore $\beta _v<0$.

To evaluate the effect of these two opposite migration strategies, we analyze the angular steady states in the two parameter regimes. Without loss of generality, we assume that, in the steady state, the mean velocity is $\bar{v}=1$. By expanding $S^1_{C_{R,n}}$ using Eq. (16), defining the components of the mean neighborhood velocity as $\bar{v}_{y,n}=\sum _{C_{R,n}\ni m\ne n }\sin \theta _m$ and $\bar{v}_{x,n}=\sum _{C_{R,n}\ni m\ne n}\cos \theta _m$, and differentiating Eq. (14), we find that the orientation of $\theta _n$ at the entropy extrema must be such that (see Supporting Information)

$$\begin{aligned} \tan \theta _n=\frac{\bar{v}_{y,n}}{\bar{v}_{x,n}}, \end{aligned}$$

but $\frac{\bar{v}_{y,n}}{\bar{v}_{x,n}}=\tan {\bar{\theta }}$, the tangent of the mean orientation of the neighbors, excluding the *n*-th cell. This results in two extremum points $\theta _n=\bar{\theta }$ and $\theta _n=\bar{\theta }+\pi$, one where the velocity of the *n*-th cell is parallel to the average velocity of its neighbors, and one when it is antiparallel. In the first case

22a $$\begin{aligned} \sin \theta _n\propto \bar{v}_{y,n} \ {\mathrm {and}} \end{aligned}$$

22b $$\begin{aligned} \cos \theta _n\propto \bar{v}_{x,n}, \end{aligned}$$

while in the second case

23a $$\begin{aligned} \sin \theta _n\propto -\bar{v}_{y,n} \ {\mathrm {and}} \end{aligned}$$

23b $$\begin{aligned} \cos \theta _n\propto -\bar{v}_{x,n}. \end{aligned}$$

It can be shown (see Supporting information) that $\theta _n=\bar{\theta }$ corresponds to an entropy minimum, while $\theta _n=\bar{\theta }+\pi$ corresponds to an entropy maximum. Consequently, the behavior of the regime $\beta _{\theta }<0$ is analogous to that of the Vicsek model^6^. Conversely, the regime $\beta _{\theta }>0$ corresponds to an anti-ferromagnetic analog of the Vicsek model.

Next, let us assume that the model has a steady state, where the Helmholtz free energy per cell is given by Eq. (7). Due to its extensivity, the Helmholtz free energy of complete, non-interacting, steady state system is

$$\begin{aligned} F_T\approx -\frac{1}{\beta _{\theta }}\sum _{n=1}^N\ln Z_n=-\frac{1}{\beta _{\theta }}\ln \left( \prod _{n=1}^NZ_n\right) , \end{aligned}$$

where $Z_n$ is the normalization constant of Eq. (3) for the *n*-th cell. For a weakly interacting system, the mean-field effective normalization constant $Z_T:=\prod _{n=1}^NZ_n$ is given by

24 $$\begin{aligned} Z_T=\int e^{-\beta _{\theta }\sum _{n=1}^N [\ln (2\pi )+\ln (1-e^{-2\gamma _n}) ]} {\mathrm {d}}\vartheta _n. \end{aligned}$$

Note that this is only valid in the limit $\beta _{\theta }\rightarrow 0$. Integrating and substituting the resulting $Z_T$ into Eq. (7) (see Supporting information), yields the Helmoltz-like free energy

25 $$\begin{aligned} F=N\left[ \left( 1-\frac{1}{\beta _{\theta }}\right) \ln (\gamma _n)+\ln (4\pi )+\frac{\ln (1-\beta _{\theta })}{\beta _{\theta }}\right] . \end{aligned}$$

Eq. (25) is well-defined only for $\beta _{\theta }<1$. This indicates that no steady state exists for $\beta _{\theta }\ge 1$, hinting at an out-of-equilibrium regime^23^. The present model belongs to the class of models with logarithmic potentials (see Eqs. (6) and (14)). The existence of a non-normalizable state in certain parameter regimes is a staple of systems with logarithmic potentials^24^.

### Collective cell migration patterns for different parameter regimes

**Figure 3:**
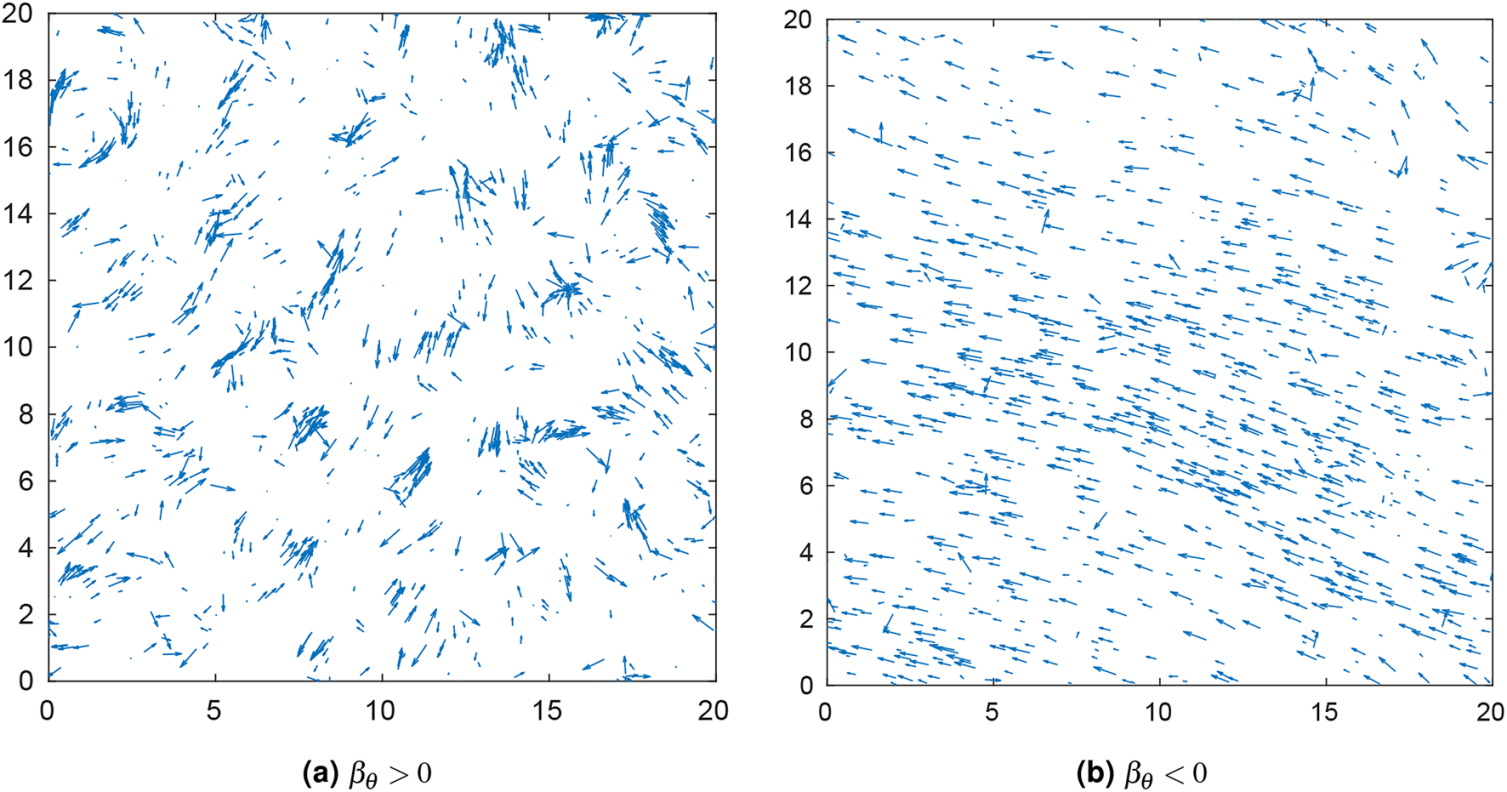
Simulation snapshots of the velocity field at long times. Arrows show the direction and magnitude of the velocity field. The snapshots were taken after 1000 time steps. 1000 particles were simulated, with an interaction radius of 3, and noise standard deviation of angles and speeds equal to 0.01. Here $g=1 , \beta _{v} = -5$ and $\varepsilon = 0$. In (**a**) the value of the angular sensitivity was equal to 18 while in (**b**) the angular sensitivity was equal to $-0.25$. Periodic boundaries were employed.

The model was implemented computationally to characterize the model and the effects of the different parameters on the resulting macroscopic behavior. The general qualitative behavior of the model can be observed in Fig. 3. In the regime $\beta _{\theta }<0$, cells tend to travel in a single direction after some time has elapsed, similar to the Vicsek model. Conversely, in the $\beta _{\theta }>0$ regime, cells are seen to move collectively in transient vortex-like structures, even after long times have elapsed. Qualitatively, the patterns resulting from different parameter combinations are summarized in Table 1. Analyzing simulations, two important phenomena are observed. First, there is a critical parametric regime $\Omega _C:=\{(\beta _{\theta },R): S_A^{1},S_A^{2}>0\}$ where patterns emerge. Specifically, for low values of interaction radius *R* no structures can be formed. This indicates that medium-to-long range spread of information is necessary for ordering. On the other hand, for $\beta _\theta >0$ for large values of *R*, outside of $\Omega _C$, again no patterns occur. This implies that for large interaction radii there is a destructive interference of the travelling information. A second important observation is that patterns do not depend on the choice of $\beta _{v}$ when this is different than zero. For $\beta _v \ne 0$, LEUP dynamics divide the population into fast and slow cells. While fast cells are useful for spreading information (and therefore, increasing the effective interaction range), slow cells are necessary for maintaining local ordering. On the other hand, if we fix the initial speed distribution and assume $\beta _{v}= 0$, then we find different patterns emerging as shown in Table 1 (also see in SI). Furthermore, we quantitatively characterized global ordering at long times. The global polar order parameter, given by Eq. (16), for the complete simulation domain, measures the global degree of polar alignment, or polarization. The global nematic order parameter, given by Eq. (17) for the complete simulation domain, measures the tendency of all cells to align nematically, or along a single axis. These order parameters take a value of one when there is global order, while taking a value of zero when the system is completely disordered. It should be noted that polar order implies nematic order, but the reverse is not true.

**Table 1:** Qualitative description of the observed patterns for different angular sensitivity and interaction radius regimes, as well as radial sensitivity.The patterning regime $\Omega _C$ is the blue area in Fig. 4a,b.

$\beta _{\theta }<0$	Radial sensitivity ($\beta _{v}$)	$R\notin \Omega _C$	$R\in \Omega _C$
	$\beta _{v} \ne 0$	Polar aligned streets of cells	Scattered polar aligned cells
	$\beta _{v} = 0$ (for uniform distribution)	Compact polar aligned cluster	Compact polar aligned cluster

$\beta _{\theta }>0$	Radial sensitivity ($\beta _{v}$)	$R\notin \Omega _C$	$R\in \Omega _C$
	$\beta _{v}\ne 0$	No order or patterns	Vortices
	$\beta _{v} = 0$ (for uniform distribution)	No order or patterns	Nematic streaming and vorticules

**Figure 4:**
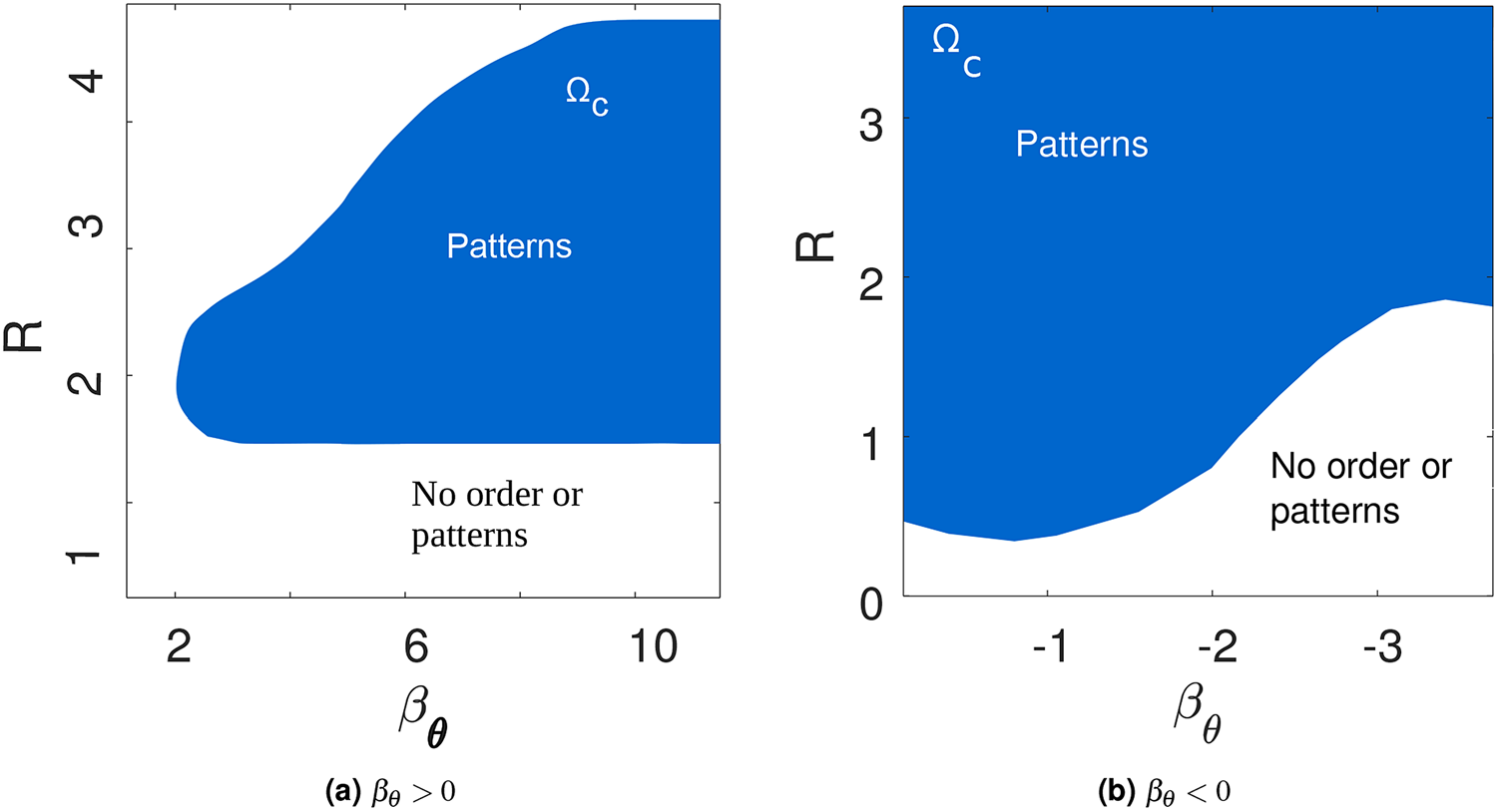
Pattern formation regimes $\Omega _C$ in the interaction radius—sensitivity plane for positive and negative values of angular sensitivity. In (**a**) there exists an optimal regime where we can find pattern formation. But in (**b**) for negative values of beta we can see patterns at smaller values of interaction radius and at smaller values of angular sensitivity.

**Figure 5:**
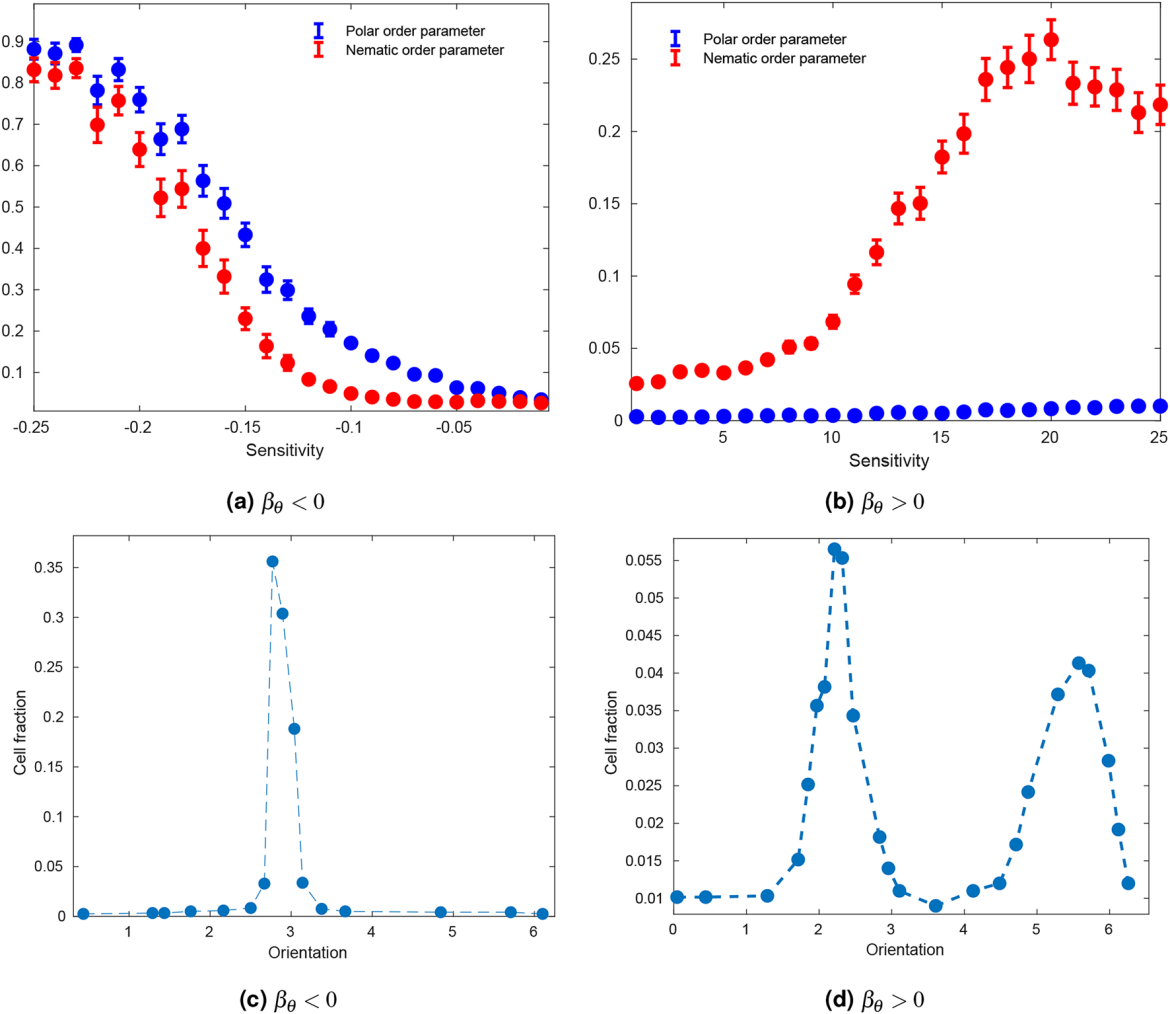
Order-disorder phase transitions and orientation distributions in two parameter regimes. Here $g=1$, $\beta _{v} = 0$, $\varepsilon = 0$ and $\langle \xi _{n}^{v}(t)^{2}\rangle =0$. (**a**) In the regime $\beta _{\theta }<0$, a phase transition towards polar order occurs at a critical value of the sensitivity. (**c**) After the phase transition, polar order arises, and all cells have roughly the same orientation. (**b**) In the regime $\beta _{\theta }>0$, the phase transition towards nematic order occurs at critical value of the sensitivity. (**d**) There is partial nematic order after the phase transition. Accordingly, several cells have opposite orientations. (**a**) and (**b**) The number of particles was fixed at $10^{3}$, noise standard deviation at 0.01, and interaction radius at 3. Values of the order parameters were averaged over 50 realizations after 1000 time steps. (**c**) and (**d**) The number of particles was fixed at 1000, noise standard deviation at 0, and interaction radius at 3. The histogram was created with data from 50 realizations after 1000 time steps.

Similarly to other velocity alignment models^7^, the model shows an order-disorder transition with increasing noise amplitude and decreasing density (SI figure Fig. 1). More importantly, we observe that in the regime $\beta _{\theta }<0$ the system also undergoes a transition towards polar order with decreasing $\beta _{\theta }$. After the transition, most particles have a similar orientation (Fig. 5a,c). In the regime $\beta _{\theta }>0$, a phase transition is also observed towards nematic order with increasing $\beta _{\theta }$. In this case, however, the nematic ordering is not perfect, as evidenced by the nematic order parameter reaching values of around 0.35 after transition (b) compared to the value of 0.9 of the polar order parameter after transition in the $\beta _{\theta }<0$ regime. This is further evidenced by the bimodal distribution of orientations with peak separation of approximately $\pi$ radians (Fig. 5d). These simulation results further corroborate our previous theoretical results.

In turn, we study the effect of speed sensitivity $\beta _v$ in terms of phase transitions. We fix the angular sensitivity $\beta _{\theta }$, either positive or negative, thus the speed distribution will only depend on $\beta _{v}$ values.

When $\beta _{v}$ is positive then speed distribution become bimodal and for $\beta _{v}<0$ the speed distribution becomes unimodal (see Fig. 10 in supplementary material). Moreover, we show that if the radial sensitivity $\beta _{v}<0$ decreases then the average speed increases. On the other hand, for any value of $\beta _{v}>0$, the first and the second moments of the speed distribution cannot be defined, since this is bimodal. Finally, for increasing cell densities, the average speed increases as well (see supplementary Fig. 9).

### A collective migration example of restricted mechanistic knowledge: the spherical bacteria case

Collective motion of bacteria has been extensively studied and modeled. Most studies have focused on the collective properties of *S. enterica*, *E. coli*, and *M. xanthus*. These species of bacteria are similar since they have a high aspect ratio. It has been shown that volume exclusion, coupled with a high aspect ratio, is sufficient to induce velocity alignment in the system^7^, and accordingly, ordered clusters of bacteria are observed at high densities.

However, it has been recently shown^25^ that even spherical *S. marcescens* bacteria do display collective migration (for experimental details please see SI section). The biophysical mechanism whereby spherical bacteria interact with one another must be different from the high body aspect ratio volume exclusion mechanism proposed for elongated bacterial species.

Recently, a combination of biophysical agent-based and hydrodynamics model has been proposed to describe these experiments. In this study the experimental observations were only partially reproduced. Therefore, the biophysical mechanisms underlying collective migration in spherical bacteria are still not well understood. An important aspect to consider is the bacterial speed $v_n$. It was found experimentally^25^ that bacterial speed followed a Rayleigh distribution, dependent on bacterial density. Collective effects on cell orientations, on the other hand, were studied by observing the vortical behavior of the population^25^.

**Figure 6:**
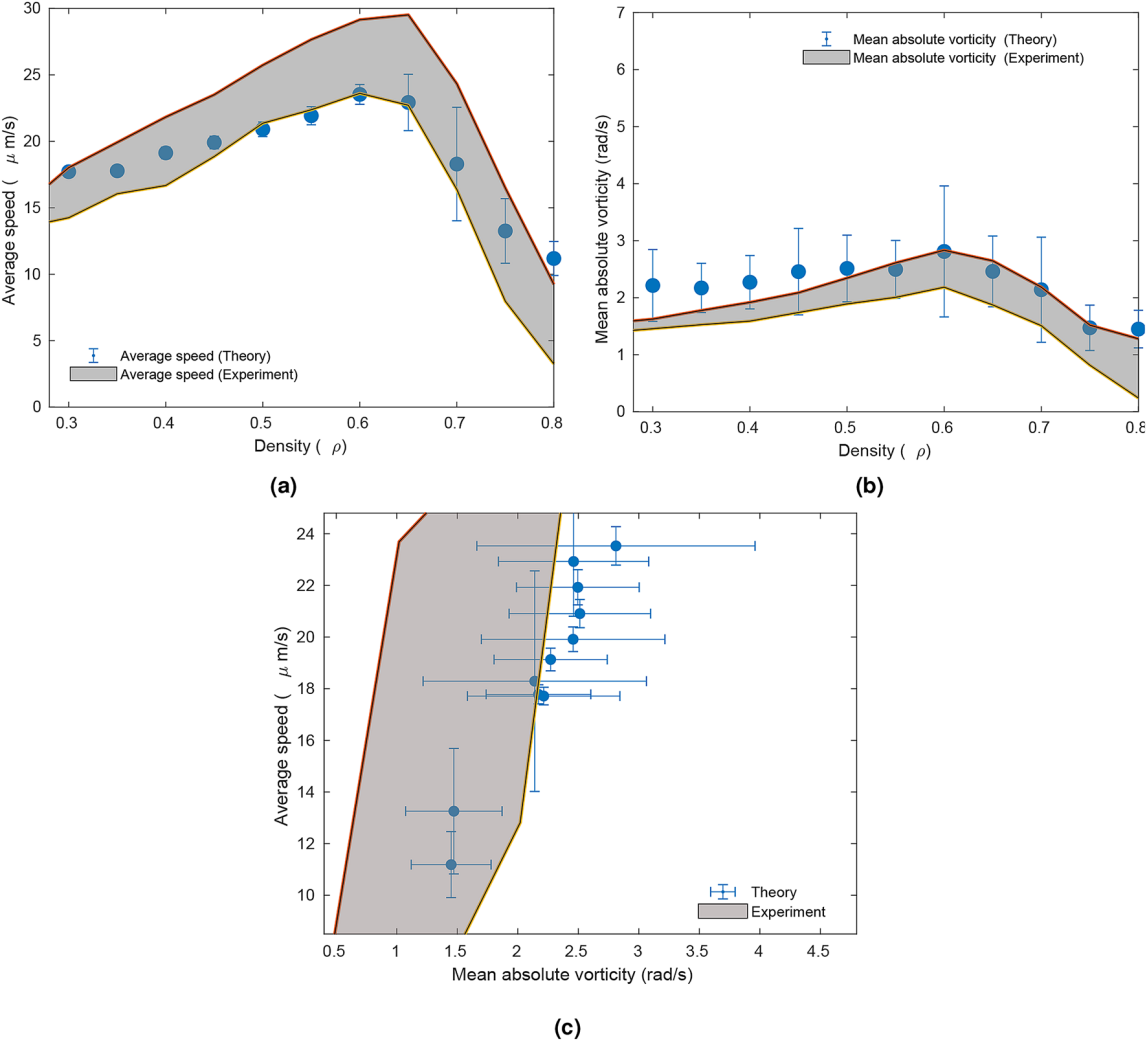
Comparison between vorticity trends in experiments and in simulations. (**a**) Relation between the average speed and the density. The simulation values shown are averaged over fifty realizations. (**b**) Dependence of the spatially normalized averaged absolute value of vorticity on the density. The simulation values shown are averaged over fifty realizations. (**c**) Relation between average speed versus mean absolute vorticity from simulations for various densities over fifty realizations. Experimental values were taken from^25^. Throughout all simulations, the standard deviation of the noise was set at 0.0001, interaction radius at $R=10$, proportionality constant $\varepsilon =0.008$, radial sensitivity $\beta _v=-20$, $g = \frac{1}{v_{n}}$ and angular sensitivity at $\beta _{\theta }=5$. Data was obtained after 500 time steps.

To reproduce the experimentally observed Rayleigh distribution for cell speed, we chose the function $g(_{v_n})=v_n^{-1}$ as shown in^16^. It is important to note that this term is not impacting the qualitative behavior of the average bacteria speed but only its variance (see SI Fig. 11). Moreover, the interested reader could see the impact of the friction term in the average cell velocity in SI Fig. 12. As shown in Fig. 6, our model qualitatively and quantitatively reproduces both the speed distribution and vorticity behavior of the experimental system. Interestingly, the behavior of the experimental system was replicated for high values of the sensitivities $\beta _v$ and $\beta _{\theta }$, and large interaction radii *R*.

Our LEUP model not only allows for a quantitative reproduction of the experiments, but also provides insight into the potential biophysical mechanisms. Such values of the sensitivities and interaction radii indicate far-reaching, strong tendencies of bacteria to average their speeds while reorienting and traveling differently from their neighbors. Spherical, rear-propelled particles have been shown to destroy polar order as a result of hydrodynamic interactions^26^, similarly to our model. Considering that *S. marcescens* is an example of a spherical, rear-propelled particle^27^, our results agree with previous findings indicating that *S. marcescens* interacts through long-range hydrodynamics^28^. The long range interaction radius suggests the existence of hydrodynamically induced interaction (which has been suggested by Ariel et. al as well as by other studies^27,28^) or self avoiding interaction^29^.

## Discussion

In this work, we have introduced an off-lattice model of LEUP-induced collective migration, based on the self-propelled particles modeling framework. It was assumed that individuals changed their radial and angular velocity components independently through LEUP. Reorientation is governed by a stochastic differential equation depending on a white noise term and a force arising from an interaction potential.

The exact form of the interaction potential can be very complex, and its specific form is dependent on particular mechanochemical details of the modeled system. While it has been shown that, in general, interactions among individuals can effectively drive the entropy of the entire system towards an extremum point^30,31^, here we do the opposite. Instead of modeling the interaction potential biophysically, it was assumed that particles followed the LEUP, which dictates that cells change their internal states in order to minimize the uncertainty of the internal states of cells in their surroundings. Although LEUP has been conceptualized to deal with high-dimensional internal states involved in cell decision-making, here we restrict on physical internal states such as speed and orientation. While cell speed was assumed to always minimize uncertainty, there was no assumption made on the cell orientation. Particles are therefore free to reorient either towards or against the gradient of entropy of the orientational distribution of particles in their neighborhood, depending on the sign of the sensitivity parameter, which also dictates the strength of the interaction. The orientational distribution in the neighborhood was assumed to be wrapped Cauchy distributed. Such a distribution facilitates the mathematical analysis of the model. However, the usage of other wrapped distributions do not qualitatively change the general behavior of the model (see SI). Please note that non-parametric methods for estimating entropies without assuming any underlying parametric distributions exist. For instance, such methods employ kernel density estimation, $k-$nearest neighbours or regression methods^32^.

We show that, when the parameter $\beta _\theta$ is negative, the model produces steady-state polar alignment patterns. Interestingly, we showed that the classical formulation of Vicsek model^6^ is a special case of LEUP. Conversely, when the parameter $\beta _\theta$ is positive, particles tend to reorient against the mean velocity of their neighborhood. In this regime, the free energy diverges, indicating an out-of-equilibrium parameter regime. This kind of parameter-dependent dichotomy is similarly observed in systems with logarithmic potentials^33^, involved in processes such as long range-interacting gases^34^, optical lattices^35^, and DNA denaturation^36^. The dichotomy arises from the logarithmic form of the entropy driving interaction in our model. It has been shown that, due to the non-normalizability of the steady state solution, such systems require a time-dependent expression for their analysis^24^. Therefore, an in-depth theoretical analysis of our model would require a similar multiparticle, time-dependent expression of the angular probability densities.

However, our LEUP migration model may go beyond the observed patterns in past Viscek-type models. In particular, in Barua et al.^37^ we have developed a discrete speed version of our LEUP migration model, where cells can have only zero or a finite speed. This model exhibits Turing patterns, i.e. dynamics clusters of non-motile cells of specific characteristic wavelength, where previously published Viscek-like models cannot may produce moving clusters of swirling cells (e.g. the milling Viscek model) but never static ones.

As a proof of principle, we show that our model replicates the collective vortical behavior of spherical motile particles. Recently, the collective behavior of spherical particles have been modeled as a combination of steric repulsion and hydrodynamic interactions^38^. Our study has shown that hydrodynamics and steric interactions induce long-range microenvironmental entropy maximization, which coincides with the $\beta _{\theta }>0$ LEUP regime. This generalizes the type of biophysical mechanisms required to produce vortical patterns.

It should be noted that, while spherical *S. marcescens* bacteria have been modeled biophysically, their collective behavior was partially reproduced^8^. This hints at an additional biological and/or biochemical interaction between cells. While our LEUP-based model is coarse-grained in terms of specific biophysical/biochemical interactions, it allows for a plausible reproduction of the experimentally observed collective velocity behavior by fitting a only few parameters. The application to spherical bacteria allows us to showcase the potential of the LEUP principle when the precise interaction mechanisms are not known.

As already mentioned, we have made some assumptions to simplify the model. Our model assumes a Gaussian, white noise term in the SDEs. This results in normal diffusive behavior in the absence of interactions. It has been observed experimentally, however, that in some conditions, cells perform Lévy walks resulting in superdiffusive behavior^39^. By changing the distribution or time correlations of the noise^9,40^, it would be possible to both replicate the non-Gaussian dynamics of single cells, and investigate the effect of single anomalous dynamics on collective behavior.

We have also assumed that particle velocities are the only internal states relevant for reorientation, for simplicity and as a proof of concept of the LEUP principle. However, it is reasonable to think that other states, such as relative position or adhesive state, may be relevant to include when modeling specific systems. This reveals an interesting point in the application of LEUP-driven models which is the selection of the most relevant/dominant internal variables. Although experimental intuition could be the easiest approach, we are currently developing a spatial principle component analysis method that would allow to select the most relevant internal variables using spatial data such as multiplexing biopsies or spatial RNA sequencing.

As stated above, LEUP circumvents the biophysical details of cell migration. The need to model systems of interacting agents without previous knowledge of the biophysical mechanisms involved has sparked at least another agent based model^41^. In this model, similarly to ours, agents act without a mechanistic rule. Rather, they consider every possible action and penalize those which are not favorable to their internal standards. While both the aforementioned model and LEUP are defined in a similar spirit, modeling under LEUP consists in correctly identifying the relevant internal cellular states for entropy optimization, while in^41^ modeling is concerned with defining suitable penalizations for each possible decision scenario.

LEUP has additional appealing features. For instance, LEUP allows for replicating a plethora of collective migration patterns. In this particular case, we have analytically derived the polar and nematic alignment Vicsek models for LEUP arguments. In this sense, LEUP acts as a generative model for collective migration mechanisms. This is particularly useful upon limited knowledge of such mechanisms, a problem called structural model uncertainty. Another advantage of LEUP is the mapping of biophysical mechanism combination to the $\beta >0$ or $\beta <0$ regimes. This allows for unifying the model analysis but for a better classification of migration mechanisms. Finally, known mechanisms or data could be easily integrated to our proposed framework by further constraining the LEUP dynamics.

## Supplementary information

Supplementary information.
