# Supplementary material for: A least microenvironmental uncertainty principle (LEUP) as a generative model of collective cell migration mechanisms
Source: Sci Rep. 2020 Dec 22;10:22371. doi: 10.1038/s41598-020-79119-y (PMC7755925; doi:10.1038/s41598-020-79119-y)
Supplement: Supplementary file 1 — Supplementary information. [file 41598_2020_79119_MOESM1_ESM.pdf]

A least microenvironmental uncertainty principle (LEUP) as a generative model of collective cell migration mechanisms.

Arnab Barua[a], **Josue M. Nava-Sedeño**[b,*], **Michael Meyer-Hermann**[a,c], **and Haralampos Hatzikirou**[a,b,**]

[a]Department of Systems Immunology and Braunschweig Integrated Centre of Systems Biology, Helmholtz Centre for Infection Research, Rebenring 56, 38106 Braunschweig, Germany
[b]Technische Univesität Dresden, Center for Information Services and High Performance Computing, Nöthnitzer Straße 46, 01062, Dresden, Germany
[c]Institute for Biochemistry, Biotechnology and Bioinformatics,Technische Universität Braunschweig, Braunschweig, Germany
[*]equal contribution
[**]Corresponding author: Haralampos Hatzikirou, hatzikirou@gmail.com

Supplementary Material

Calculation of the curvature of micro-environmental entropy and Helmholtz free energy

The entropy of the wrapped Cauchy distribution is

$$
\begin{aligned}
S(\Theta_n \mid \theta_n) &= \ln(2\pi) \\
&+ \ln\left\{1 - \frac{1}{N_{C_{R,n}}^2}\left[\bar{v}_{y,n}^2 + \bar{v}_{x,n}^2 + 2\left(\bar{v}_{y,n}\sin\theta_n + \bar{v}_{x,n}\cos\theta_n\right)\right]\right\}.
\end{aligned} \tag{1}
$$

To discriminate the maximum from the minimum point, the value of $\frac{\partial^2}{\partial \theta_n^2} S(\Theta_n \mid \theta_n)$ must be evaluated at each extremum point. The second derivative is given by

$$
\begin{aligned}
\frac{\partial^2}{\partial \theta_n^2} S(\Theta_n \mid \theta_n) =& \frac{\frac{2}{N_{C_{R,n}}^2}\left(\bar{v}_{y,n}\sin\theta_n + \bar{v}_{x,n}\cos\theta_n\right)}{1 - \frac{1}{N_{C_{R,n}}^2}\left[\bar{v}_{y,n}^2 + \bar{v}_{x,n}^2 + 2\left(\bar{v}_{y,n}\sin\theta_n + \bar{v}_{x,n}\cos\theta_n\right)\right]} \\
&- \left\{\frac{\frac{2}{N_{C_{R,n}}^2}\left(\bar{v}_{y,n}\cos\theta_n - \bar{v}_{x,n}\sin\theta_n\right)}{1 - \frac{1}{N_{C_{R,n}}^2}\left[\bar{v}_{y,n}^2 + \bar{v}_{x,n}^2 + 2\left(\bar{v}_{y,n}\sin\theta_n + \bar{v}_{x,n}\cos\theta_n\right)\right]}\right\}^2.
\end{aligned}
$$

In an extremum point, $\frac{\partial}{\partial \theta_n} S(\Theta_n \mid \theta_n) = 0$, therefore

$$
\left.\frac{\partial^2}{\partial \theta_n^2} S(\Theta_n \mid \theta_n)\right|_{\text{ext}} = \frac{\frac{2}{N_{C_{R,n}}^2}\left(\bar{v}_{y,n}\sin\theta_n + \bar{v}_{x,n}\cos\theta_n\right)}{1 - \frac{1}{N_{C_{R,n}}^2}\left[\bar{v}_{y,n}^2 + \bar{v}_{x,n}^2 + 2\left(\bar{v}_{y,n}\sin\theta_n + \bar{v}_{x,n}\cos\theta_n\right)\right]}. \tag{2}
$$

Defining κ as the proportionality constant relating $\sin\theta_n$ and $\cos\theta_n$ with $\bar{v}_{y,n}$ and $\bar{v}_{x,n}$, respectively, the second derivative evaluated at $\theta_n = \bar{\theta}$ is

$$
\left.\frac{\partial^2}{\partial \theta_n^2} S(\Theta_n \mid \theta_n)\right|_{\theta_n=\bar{\theta}} = \frac{\frac{2\kappa}{N_{C_{R,n}}^2}\left(\bar{v}_{y,n}^2 + \bar{v}_{x,n}^2\right)}{1 - \left(S_{C_{R,n}}^1\right)^2} > 0, \tag{3}
$$

because the numerator is positive definite, and the denominator is positive given the bounds of the order parameters. The extremum point $\theta_n = \bar{\theta}$ therefore corresponds to an entropy minimum. Consequently, the behavior of the regime $\beta < 0$ is analogous to that of the Vicsek model. Conversely, at $\theta_n = \bar{\theta} + \pi$ we find that

$$
\left.\frac{\partial^2}{\partial \theta_n^2} S(\Theta_n \mid \theta_n)\right|_{\theta_n=\bar{\theta}} = \frac{\frac{-2\kappa}{N_{C_{R,n}}^2}\left(\bar{v}_{y,n}^2 + \bar{v}_{x,n}^2\right)}{1 - \left(S_{C_{R,n}}^1\right)^2} < 0, \tag{4}
$$

using the same arguments as for the $\theta_n = \bar{\theta}$ point. Therefore, the point $\theta_n = \bar{\theta} + \pi$ corresponds to the entropy maximum. Then, the regime $\beta > 0$ corresponds to a nematic analog of the Vicsek model. Next, let us assume that the model has a steady state, where the Helmholtz free energy per bacterium is given by $F = -\frac{1}{\beta_\theta}\ln Z$. Due to its extensivity, the Helmholtz free energy of the complete system is

$$
F_T = -\frac{1}{\beta_\theta}\sum_{n=1}^{N}\ln Z_n = -\frac{1}{\beta_\theta}\ln\left(\prod_{n=1}^{N} Z_n\right),
$$

where Z_n is the normalization constant of n-th cell(see Eq.(3) in paper).

The effective normalization constant $Z_T := \prod_{n=1}^{N} Z_n$ is given by

$$
Z_T = \int e^{-\beta_\theta \sum_{n=1}^{N}\left[\ln(2\pi) + \ln\left(1 - e^{-2\gamma_n}\right)\right]} \mathrm{d}\vartheta_n. \tag{5}
$$

The integration is performed over the orientations of all cells in the system. Moreover, the dependency of each γ_n on all angles θ_n is complex and makes integration challenging. However, variation of θ_n for all n translates into a variation in all γ_n. Therefore, Eq. 5 is equivalent to

$$Z_T = \int e^{-\beta_\theta \sum_{n=1}^{N} \left[\ln(2\pi)+\ln\left(1-e^{-2\gamma_n}\right)\right]} \mathrm{d}\gamma_n. \tag{6}$$

Expanding up to linear terms around $\gamma_n = 0$ yields

$$Z_T = \int e^{-\beta_\theta \sum_{n=1}^{N} [\ln(2\pi)+\ln(2\gamma_n)]} \mathrm{d}\gamma_n,$$

which after rearranging terms and integrating reduces to

$$Z_T = \left[\frac{1}{(4\pi)^{\beta_\theta}} \frac{\gamma_n^{1-\beta_\theta}}{1-\beta_\theta} \right]^N. \tag{7}$$

Substituting Eq. 7 into the expression of the Helmholtz free energy (Eq. (6) in the main text), and rearranging terms, yields the Helmholtz free energy

$$F = N \left[\left(1 - \frac{1}{\beta_\theta} \right) \ln(\gamma_n) + \ln(4\pi) + \frac{\ln(1-\beta_\theta)}{\beta_\theta} \right]. \tag{8}$$

Eq. 8 is well-defined only for $\beta_\theta < 1$. This indicates that no steady state exists for $\beta_\theta \geq 1$, hinting at an out-of-equilibrium regime. The present model belongs to the class of models with logarithmic potentials.
The existence of a non-normalizable state in certain parameter regimes is a staple of systems with logarithmic potentials.

Pattern formation in different β_θ regime (see Table 1) ($\beta_v = 0$)

We have defined the range of the polar order parameter as P, nematic order parameter as N and mean absolute vorticity as V.

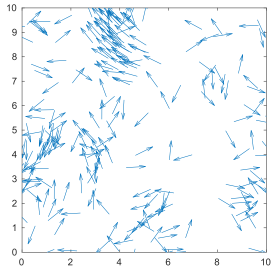

(a) Polar aligned street cells (P=0.6 to 0.7, N=0.5 to 0.7, V=0 to 0.04)

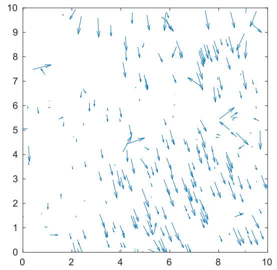

(b) Compact polar aligned cells (P=0.8 to 1.0, N=0.7 to 1.0, V=0 to 0.065)

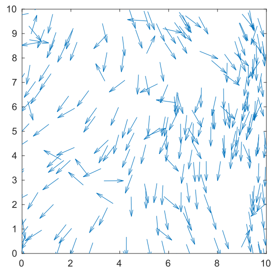

(c) Scattered polar aligned cells (P=0.7 to 1.0, N=0.7 to 1.0, V=0 to 0.05)

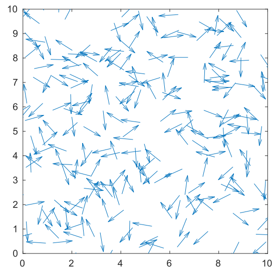

(d) No order or patterns (P=0 to 0.09, N=0 to 0.07, V=0 to 0.065)

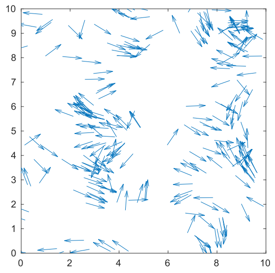

(e) Nematic streaming (P=0 to 0.05, N=0.3 to 0.5, V=0 to 0.065)

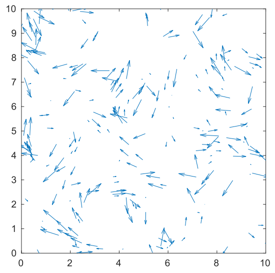

(f) Nematic streaming and vorticules (P=0 to 0.03, N=0.2 to 0.4, V=0 to 0.04)

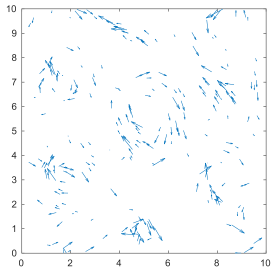

(g) Vortices (P=0 to 0.03, N=0.2 to 0.4, V=0.1 to 0.35)

Figure 1. All type of patterns are captured for different β_θ values. Patterns have been changed due to velocity distributions and interaction radius.

Polar order parameter in angular sensitivity ($\beta_\theta < 0$) regime

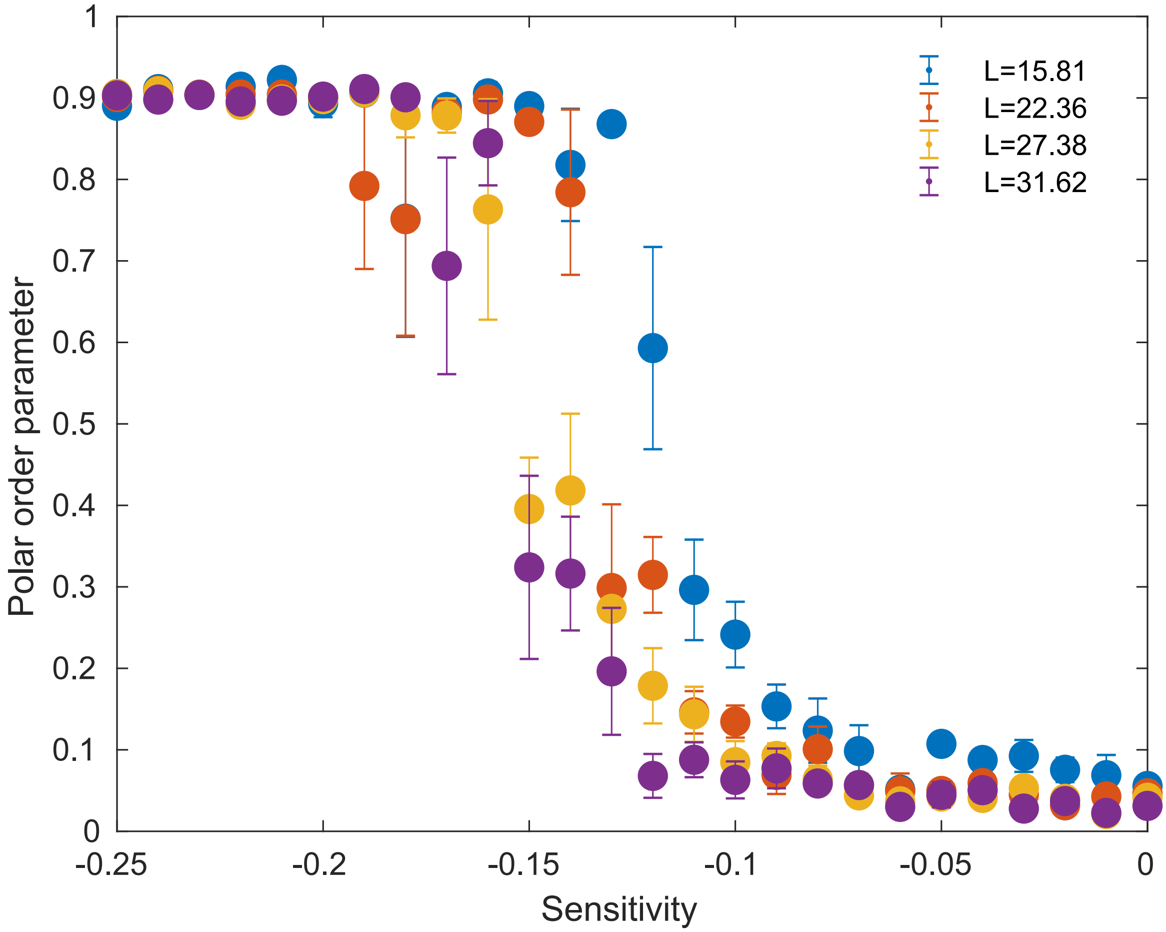

Figure 2. Polar order parameter vs. angular sensitivity graph. Where interaction radius is at 3, and standard deviation of noise is at 0.1. Density is fixed at 1.0. Here micro-environmental entropy has been taken from wrapped Cauchy distribution. Here $g = 1, \beta_v = 0$, $\varepsilon = 0$ and $\langle \xi_n^v(t)^2 \rangle = 0$. All the order parameters were averaged over 5 realizations after 10^3 time steps.

Polar order parameter vs. angular noise graph

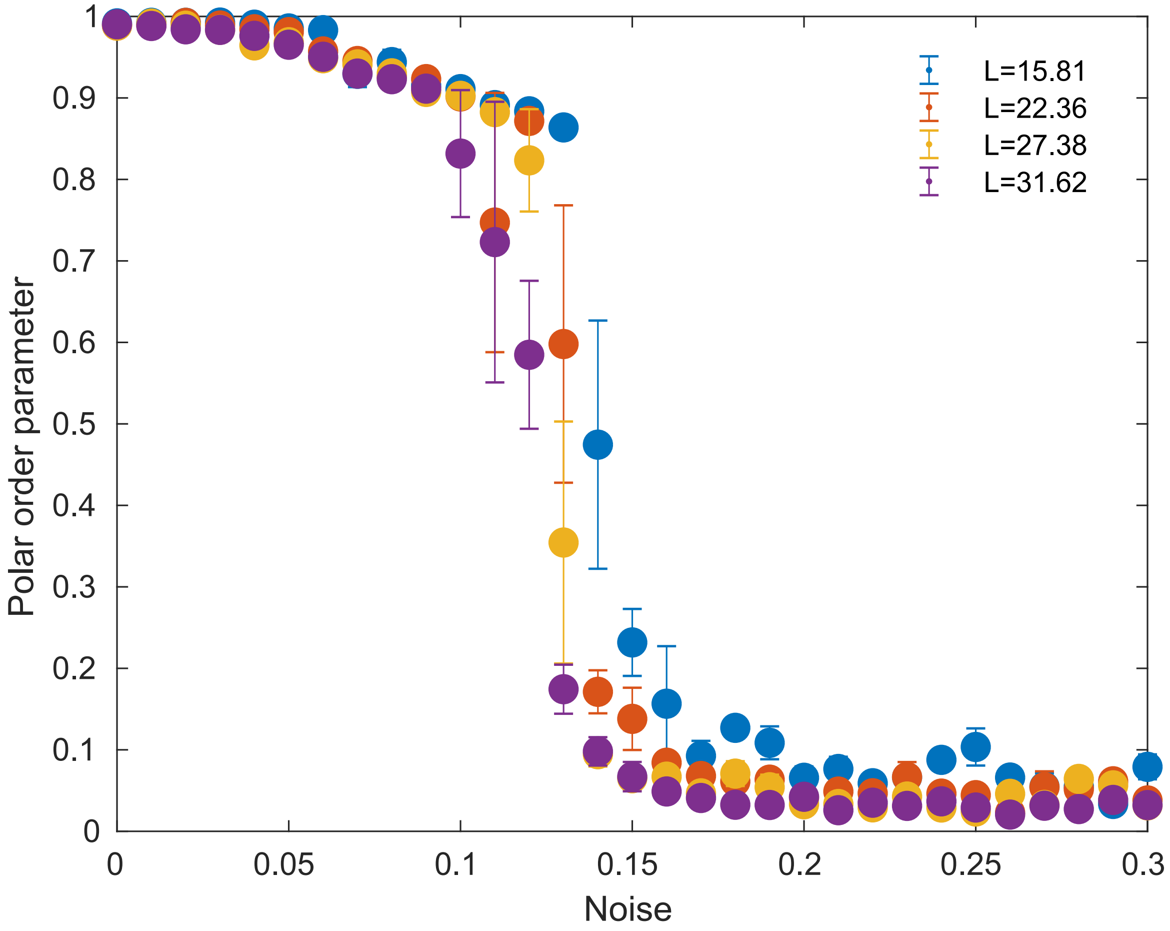

Figure 3. Polar order parameter vs. angular noise graph. Where interaction radius is at 3 and angular sensitivity is at -0.2. Density is fixed at 1.0. Here micro-environmental entropy has been taken from wrapped Cauchy distribution. Here $g = 1, \beta_v = 0$, $\varepsilon = 0$ and $\langle \xi_n^v(t)^2 \rangle = 0$. All the order parameters were averaged over 5 realizations after 10^3 time steps.

Nematic order parameter in angular sensitivity ($\beta_\theta > 0$) regime

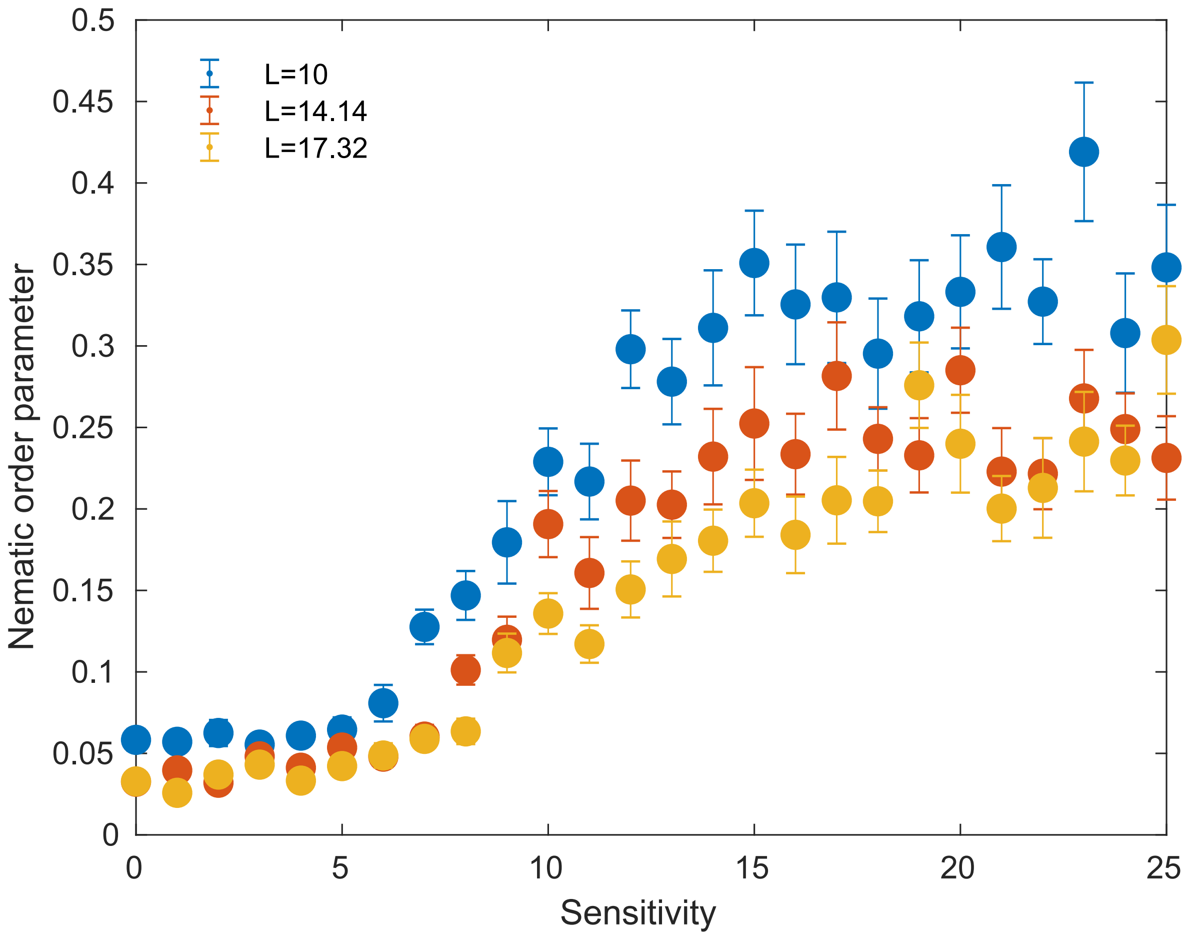

Figure 4. Nematic order parameter vs. angular sensitivity graph. Where interaction radius is at 3 and standard deviation of angular noise is at 0.05. Density is fixed at 2.5. Here micro-environmental entropy has been taken from wrapped Cauchy distribution. Here $g = 1, \beta_v = 0$, $\varepsilon = 0$ and $\langle \xi_n^v(t)^2 \rangle = 0$. All the order parameters were averaged over 20 realizations after 10^3 time steps.

Nematic order parameter vs. angular noise graph

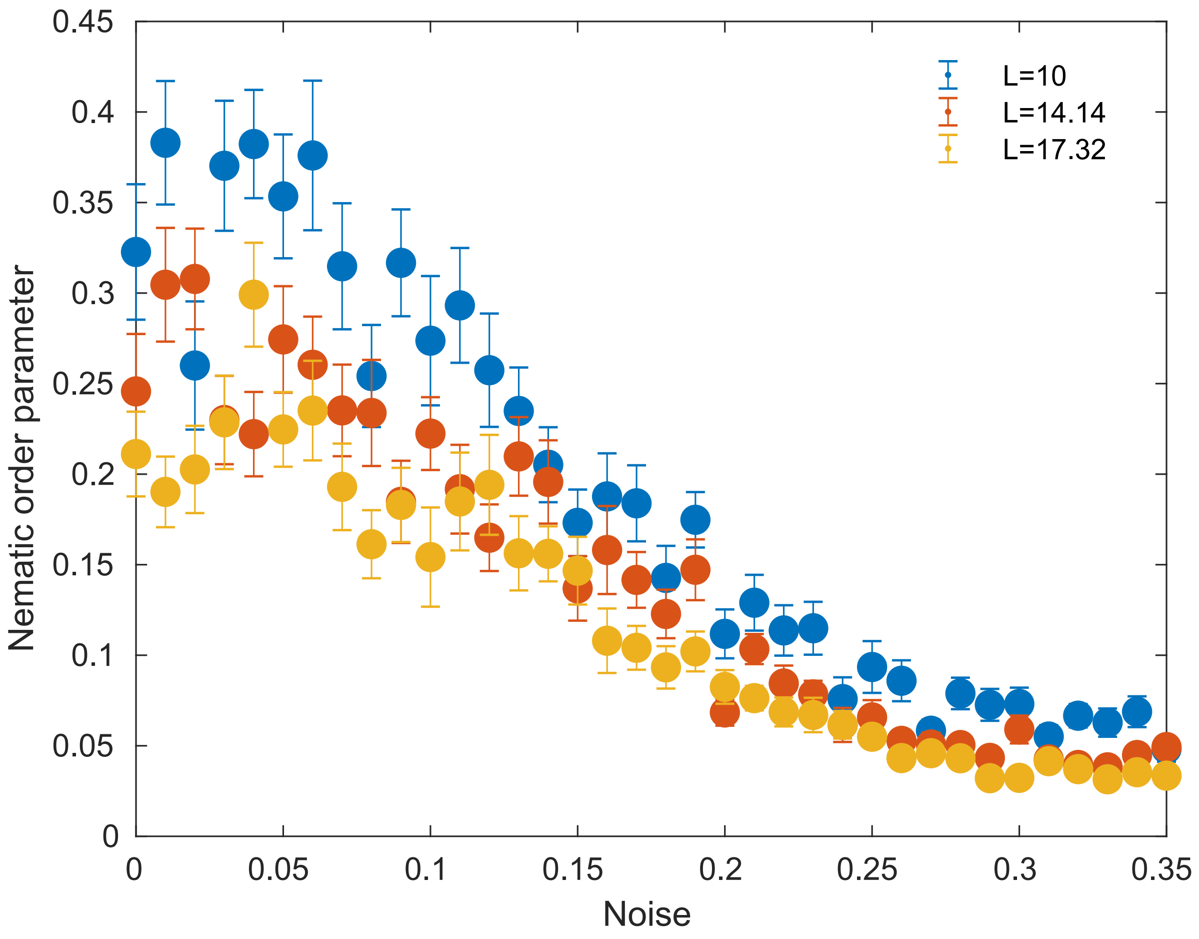

Figure 5. Nematic order parameter vs. angular noise graph. Where interaction radius is at 3 and sensitivity is at 20.Density is fixed at 2.5. Here micro-environmental entropy has been taken from wrapped Cauchy distribution. Here $g = 1, \beta_v = 0$, $\varepsilon = 0$ and $\langle \xi_n^v(t)^2 \rangle = 0$. All the order parameters were averaged over 20 realizations after 10^3 time steps.

Order parameters vs. density graph

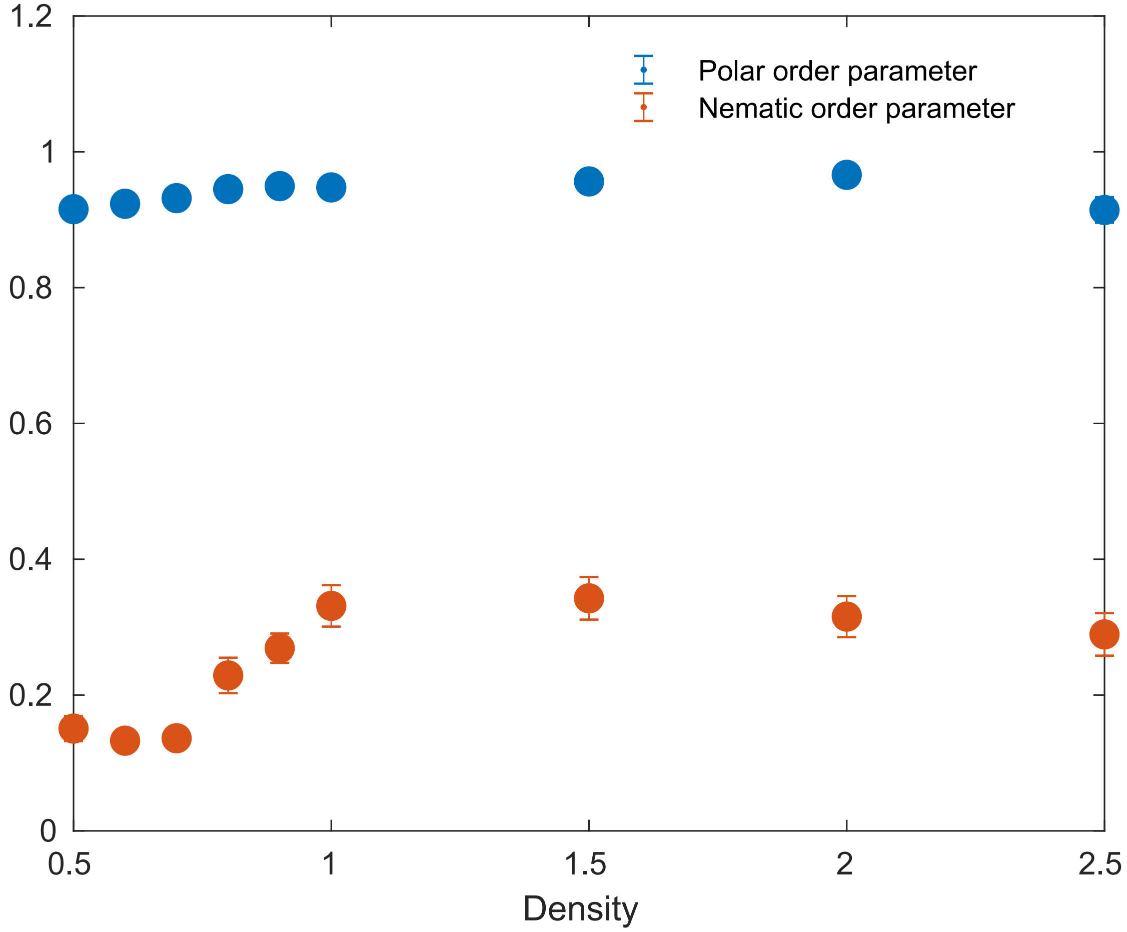

Figure 6. Polar order parameter and nematic order parameter vs. density graph. Where interaction radius is at 3 and sensitivity is fixed at 15(for nematic order parameter) and -0.2 (for polar order parameter). Here micro-environmental entropy has been taken from wrapped Cauchy distribution. Here $g = 1, \beta_v = 0$, $\varepsilon = 0$ and $\langle \xi_n^v(t)^2 \rangle = 0$. All the order parameters were averaged over 20 realizations after 10^3 time steps.

Polar order parameter and nematic order parameter in angular sensitivity ($\beta_\theta < 0$) regime

When we took micro-environmental entropy from wrapped exponential distribution we didn't find any qualitative change in phase transition phenomena in $\beta_\theta < 0$ regime.

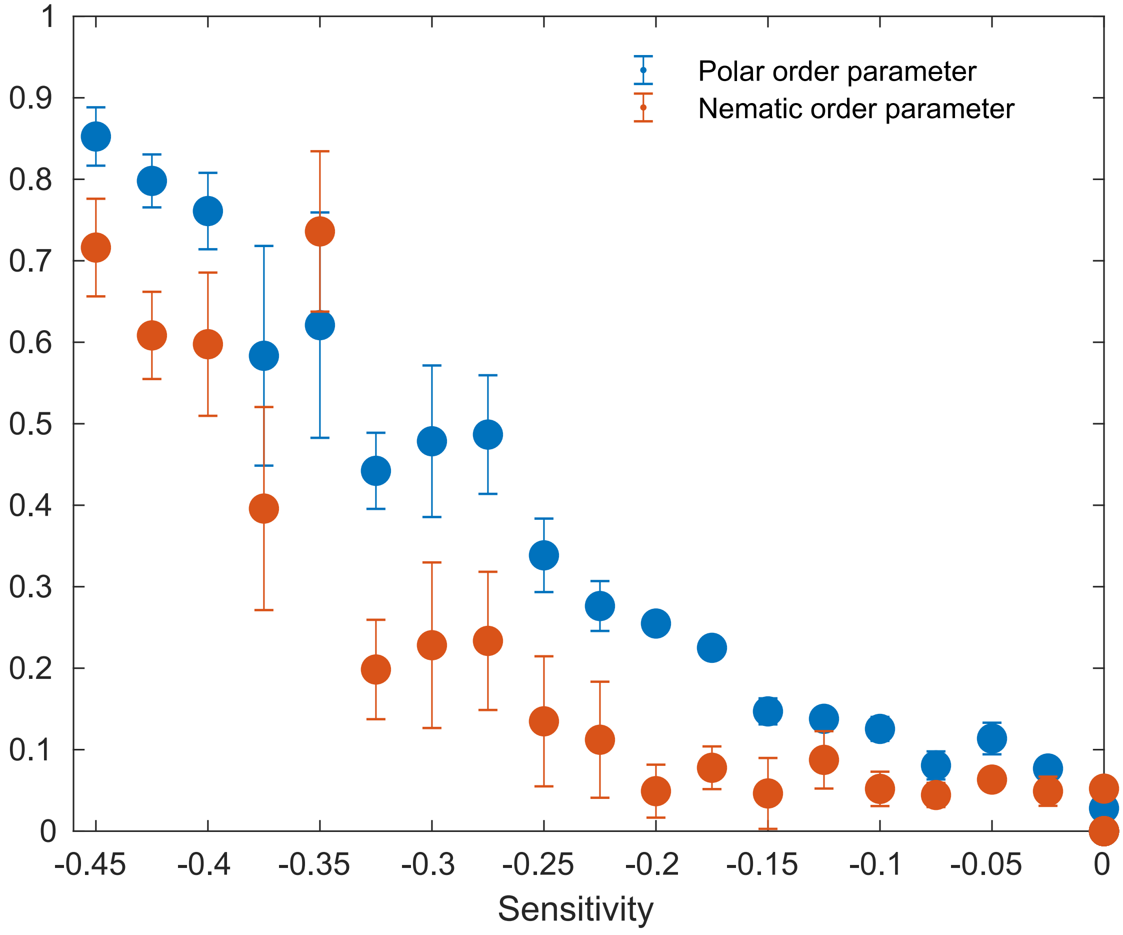

Figure 7. Polar order parameter and nematic order parameter vs. angular sensitivity ($\beta_\theta < 0$) graph. Where interaction radius is at 3, and standard deviation of noise is at 0.05. Density is fixed at 2.5. Here micro-environmental entropy has been taken from wrapped exponential distribution. Here $g = 1, \beta_v = 0$, $\varepsilon = 0$ and $\langle \xi_n^v(t)^2 \rangle = 0$. All the order parameters were averaged over 5 realizations after 250 time steps.

Polar order parameter and nematic order parameter in angular sensitivity ($\beta_\theta > 0$) regime

When we took micro-environmental entropy from wrapped exponential distribution, we didn't find any qualitative change in phase transition phenomena in $\beta_\theta > 0$ regime.

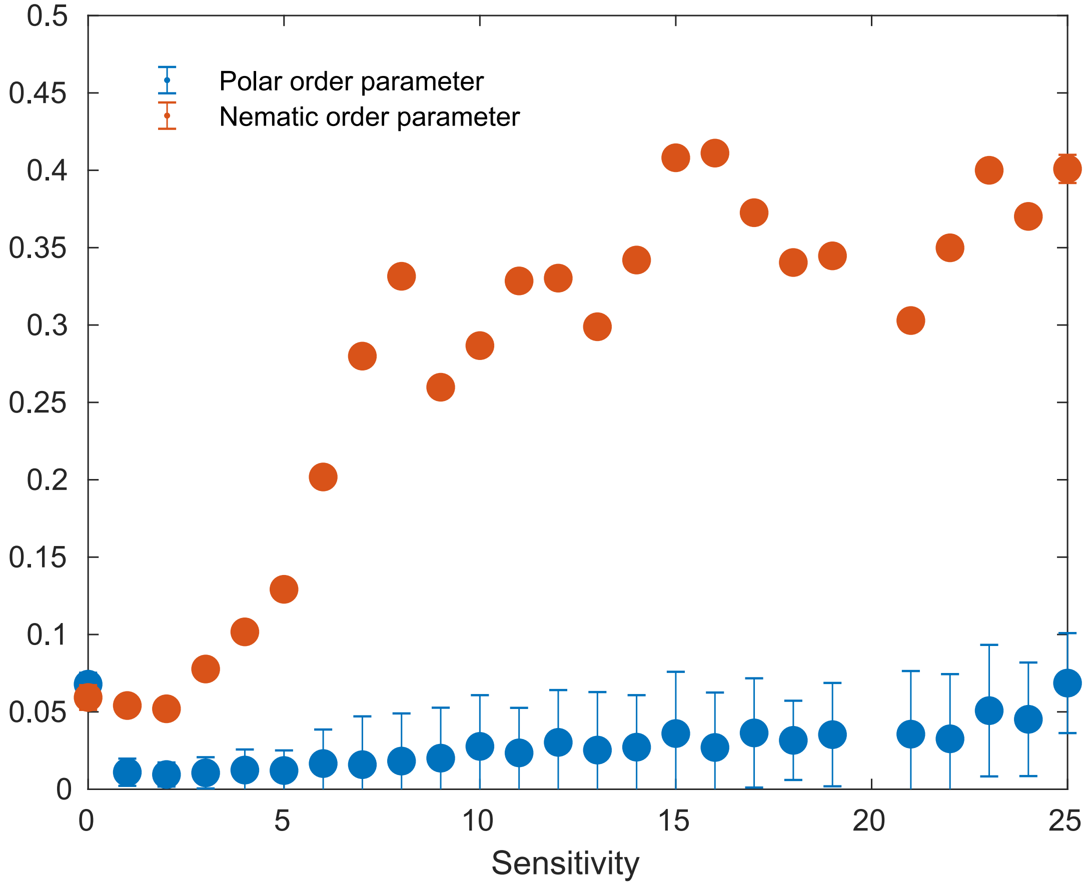

Figure 8. Polar order parameter and nematic order parameter vs. angular sensitivity ($\beta > 0$) graph. Where interaction radius is at 3, and standard deviation of noise is at 0.05. Density is fixed at 2.5. Here micro-environmental entropy has been taken from wrapped exponential distribution. Here $g = 1, \beta_v = 0$, $\varepsilon = 0$ and $\langle \xi_n^v(t)^2 \rangle = 0$. All the order parameters were averaged over 20 realizations after 10^3 time steps.

Average speed vs. radial sensitivity

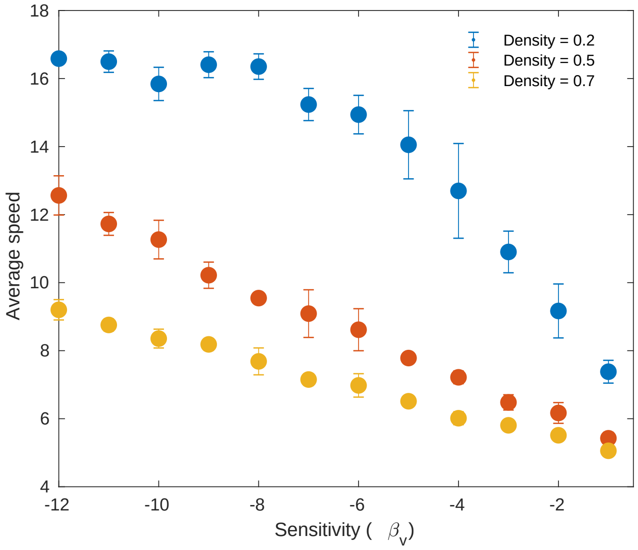

Figure 9. Average speed vs. radial sensitivity (β_v) for different densities. The standard deviation of angular noise was fixed at 0.01, $\beta_\theta = 20$, $\psi = 0$, the box size has been fixed to 30 and the interaction radius was $R = 10$.

Probability distributions for speed

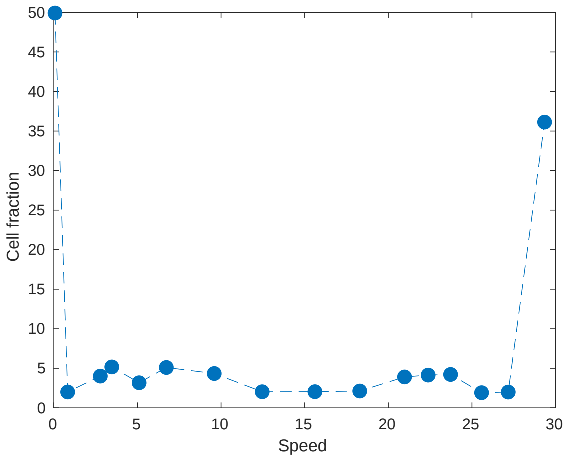

(a) $\beta_v > 0$

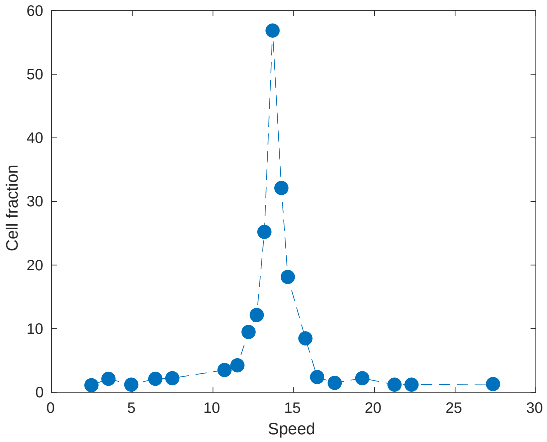

(b) $\beta_v < 0$

Figure 10. The number of particles was fixed at 200, noise standard deviation at 0.05, β_θ is at -2 and interaction radius at 4. For (a) and (b) β_v were fixed at 25 and -10. Simulations were averaged over 15 realizations after 200 time steps.

Average speed vs. density graph (depends on multiplicative noise)

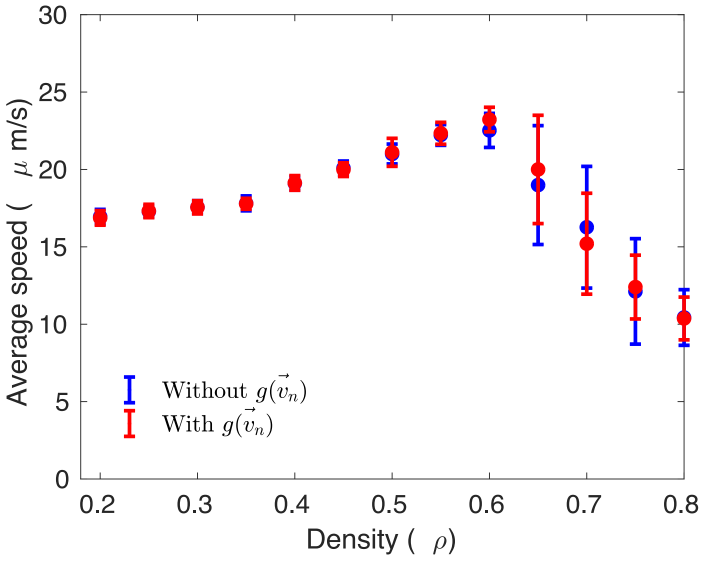

Figure 11. Difference of average speed simulations including or not the multiplicative noise term $g(\vec{v_n})$
.

Averagve value of the friction term vs density graph

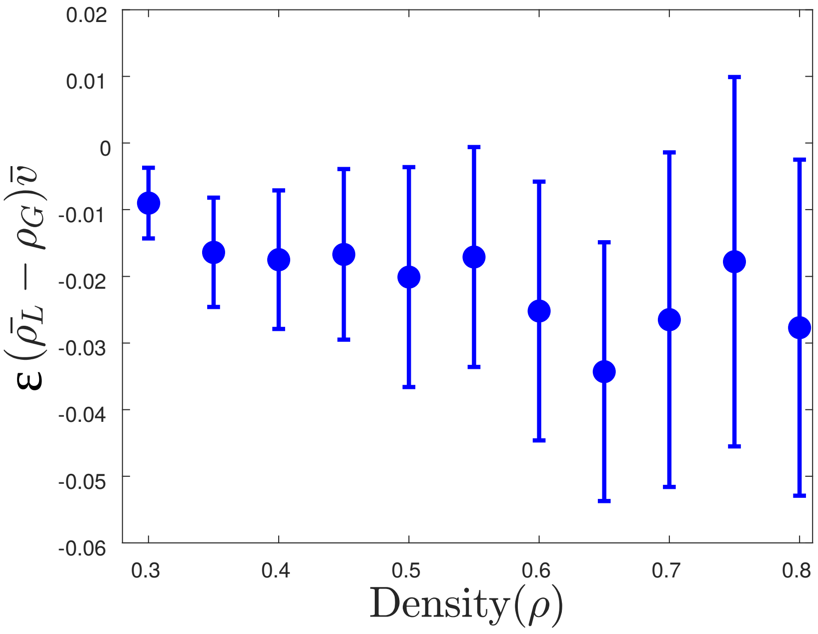

Figure 12. Average value of the friction term in different densities. Throughout all simulations, the standard deviation of the noise was set at 0.0001, interaction radius at $R = 10$, proportionality constant $\varepsilon = 0.008$, radial sensitivity $\beta_v = -20$, $g = \frac{1}{v_n}$ and angular sensitivity at $\beta_\theta = 20$. Data was obtained after 500 time steps.

Details on the experimental setup

The experiment[1] has been done with gram negative bacteria Serratia marcescens 274. In the exponential growth phase at low density (i.e. 1×10^7 cells) the geometry of bacteria was rod shaped but due to starvation the geometry of the bacteria become spherical. The bacteria were grown to an OD650 of 2.0, corresponding to approximately 2×10^9 bacteria/ml. A 5-µl drop of an overnight (18 h) WT S. marcescens 274 culture have been placed on the glass slide. The density of the bacteria was fixed at 2×10^9 cells/ml initially and the aspect ratio was smaller than 1.1. Cells were coming and swimming on the upper surface of the drop. Surface density were increased from minimal to maximal lasted approximately 20 min. The motion of the bacteria was independent of geometry of the drop, buyoancy and gravity.

References

1. Rabani, A., Ariel, G. & Be'er, A. Collective motion of spherical bacteria. *PloS one* **8**, e83760 (2013).
